# Cholecystectomy-induced secondary bile acids accumulation ameliorates colitis through inhibiting monocyte/macrophage recruitment

**DOI:** 10.1080/19490976.2022.2107387

**Published:** 2022-09-01

**Authors:** Yun Liu, Jun Xu, Xinhua Ren, Yu Zhang, Ziliang Ke, Jianhua Zhou, Yang Wang, Yifan Zhang, Yulan Liu

**Affiliations:** a Department of Gastroenterology, Peking University People’s Hospital, Beijing, China; b Clinical Center of Immune-Mediated Digestive Diseases, Peking University People’s Hospital, Beijing, China; c Center of Liver Diseases, Beijing Ditan Hospital, Capital Medical University, Beijing, China; d Institute of Clinical Molecular Biology & Central Laboratory, Peking University People’s Hospital, Beijing, China

**Keywords:** post-cholecystectomy, inflammatory bowel disease, bile acids, microbiota, macrophages

## Abstract

Although post-cholecystectomy (PC) patients usually have gastrointestinal complications and a higher risk of colorectal cancer, previous studies undetected a heightened risk of inflammatory bowel disease. Thus, we tried to investigate cholecystectomy’s impact and pathophysiological mechanism on murine colitis models and clarify the association among fecal bile acids (BAs), mucosal bacterial microbiota, and immune cells in the PC patients. One month or three months after cholecystectomy, mice have induced colitis and tested BAs and fecal microbiota analysis. Next, mice were treated with various cholecystectomy-accumulated bile acids in drinking water for three months before inducing colitis. All 14 paired PC patients and healthy subjects were enrolled for BAs and mucosal microbiota analysis. Cholecystectomy ameliorated DSS-induced murine colitis, accelerated mucosal repair, and induced a significant shifting of fecal microbiota and BAs profiles under colitis status, which featured a higher relative abundance of species involved in BAs metabolism and increased secondary BAs concentrations. Cholecystectomy-associated secondary BAs (LCA, DCA, and HDCA) also ameliorated DSS-induced colitis and accelerated mucosal repair in mice. Cholecystectomy and specific secondary BAs treatments inhibited monocytes/macrophages recruitment in colitis mice. *In vitro*, cholecystectomy-associated secondary BAs also downregulated monocytes chemokines in the THP-1 derived macrophages through activation of the LXRα-linked signaling pathway. The alterations of mucosal microbiota and fecal BAs profiles were found in the PC patients, characterized as increased species with potential immuno-modulating effects and secondary BAs, which were negatively associated with peripheral monocytes levels. Cholecystectomy-induced secondary bile acids accumulation ameliorated colitis through inhibiting monocyte/macrophage recruitment, which might be mediated by the LXRα-related signaling pathway. Cholecystectomy, after 3 months follow-up, has an immune-regulatory role in murine colitis, preliminarily explaining that no increased risk of IBD had been reported in the PC patients, which still warrants further studies.

## Introduction

Cholecystectomy is the most common operation in biliary surgery; however, post-cholecystectomy (PC) patients have higher risks of post cholecystectomy syndrome and even colorectal cancer (CRC) in long-term outcomes.^1,2^ The persistence of gastrointestinal symptoms following cholecystectomy (post-cholecystectomy syndrome) may occur in 5 ~ 47% of patients.^2,3^ Cholecystectomy also induced intestinal bacterial and bile acids dysbiosis.^4,5^ Even so, few studies reported the association between cholecystectomy and IBD. In a population-based cohort study, the risk of IBD did not change significantly between the PC patients and healthy controls (0.062% vs 0.051%).^6^ The mentioned results are limited to illustrating the role of cholecystectomy in the occurrence of IBD.

The gallbladder, a reservoir for concentrating and storing bile, allows for 20 ~ 30% emptying during fasting, while 70 ~ 80% emptying after a meal.^7^ Cholecystectomy removes this pacemaker from the enterohepatic circulation and continuously secretes the bile into the intestinal tract. Previous studies reported that the bile acids (BAs) pool had a regular or small size while circulating more quickly after cholecystectomy.^8,9^ Consequently, increased secondary BAs concentrations were found in the PC patients.^10–13^ Cholecystectomy also induced intestinal bacterial dysbiosis, such as an abundant increase of *Fusobacterium, Bacteroides*, and *Escherichia*, which was associated with gastrointestinal dysfunction.^14–18^ These results preliminarily implied cholecystectomy changed BAs and microbiota in the colon, but these alterations’ roles had not been widely investigated in IBD patients.

As we all know, the interaction between BAs and intestinal microbiota is bidirectional and highly complex in the IBD pathogenesis.^19^ Gut microbiota is involved in BAs metabolism and affects the composition of BAs. Inversely, the altered BAs profiles could further reshape bacterial microbiota.^19^ Accumulating evidence has shown that gut microbiota dysbiosis is one of the primary triggers in IBD, while its molecular mechanisms and mediators have not been fully understood.^20^ Recently, BAs and their receptors (BARs), which interact between the host and intestinal microbiota, have been investigated in the IBD patients.^21^ BAs profile of the IBD patients showed increased chenodeoxycholic acid (CDCA) levels and decreased secondary BAs concentrations, such as lithocholic acid (LCA) and deoxycholic acid (DCA).^22^ Genomic analysis also implied the depletion of BA biotransformation and production capabilities in the microbiota of IBD patients.^23^

Furthermore, several mucosal immune cells (such as monocytes, macrophages, dendritic cells, and T cells) can be activated by BAs and exert immune regulatory effects.^22^ A recent study found that DCA and LCA alleviated inflammation in murine colitis models.^24^ It is worth noting that high-dose and long-term DCA treatment could aggravate intestinal inflammation and accelerate the transition from intestinal adenoma to colonic adenocarcinoma.^25,26^ Based above, gut microbiota, BAs, and BARs have complex cooperation to affect the IBD development, which still needs further investigations.

Cholecystectomy is one of the crucial factors, which affects BAs-gut microbiota crosstalk, and its effect on IBD pathogenesis has not yet been elucidated. Our data indicated that cholecystectomy-induced secondary BAs accumulation ameliorated colitis through inhibiting monocyte/macrophage recruitment, which the LXRα-related signaling pathway might mediate. The underlying mechanism showed BAs regulated inflammatory homeostasis and preliminarily explained that PC patients had not been reported increased IBD risk.

## Results

### Cholecystectomy ameliorates DSS-induced murine colitis

**Figure 1.**
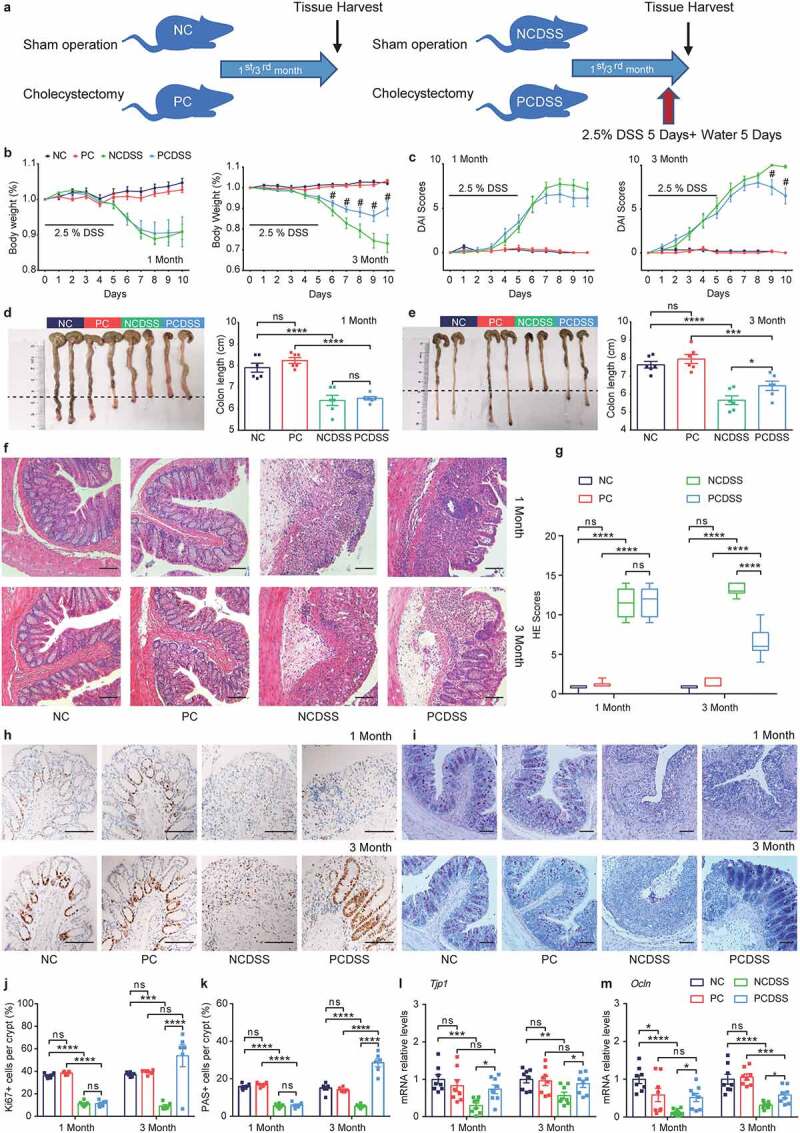
Cholecystectomy ameliorates DSS-induced murine colitis. a. Schematic diagram showing the overall design and complete timeline. Mice were induced colitis by DSS treatment at the first or third month after cholecystectomy. b. Body weight change (relative to starting weight, set as 100%) during the course of DSS-induced colitis. c. Disease activity index during colitis models. d-e. Representative colonic images and colon length. f-g. Representative hematoxylin and eosin-stained (f) sections and histological scores (g) of colons. h-i. Immunohistochemical staining of Ki67+ and Periodic Acid-Schiff staining (PAS)+ in colon sections. J-k. Quantitative analysis of colonic ki67+ and PAS+ cells. l-m. Relative mRNA expression of *Tjp1* (l) and *Ocln* (m) in colon tissues. Scale bar, 100 μm. Data are represented as mean ± SEM. N = 6–8 per group. *P < .05, **P < .01, ***P < .001, ****P < .0001, ^#^P < .05 compared with NCDSS. ns: not significant. DSS, dextran sulfate sodium; HE, hematoxylin & eosin; NC, normal control; NCDSS, normal control with DSS; Ocln, occluding; PC, Cholecystectomy; PCDSS, Cholecystectomy with DSS; Tjp1, tight junction protein 1.

Based on our published results, bacterial dysbiosis after cholecystectomy was more obvious with the increase of the duration after cholecystectomy.^17^ We performed cholecystectomy murine models and induced colitis at the first or third month after the operation as described above (Figure 1a) to determine whether cholecystectomy could affect colitis development with the time increased. The PC mice appeared average body weight gain compared with the NC mice during experimental observation (Figure S1a). When the mice challenged DSS at the first month after cholecystectomy (PCDSS) or sham operation (NCDSS), the two groups showed no differences in the signs of inflammation or mucosal repair (Figure 1b-k). While the decreased mRNA levels of tight junction protein (ZO1, Occludin) caused by DSS were relieved in the PCDSS mice (Figure 1l,m). Interestingly, at the 3^rd^ month, the DSS treatment induced mild colitis in the PCDSS mice, as exemplified by less weight lost (Figure 1b), lower DAI scores (Figure 1c), longer colon length (Figure 1d,e), and lower histological scores (Figure 1f,g). Simultaneously, the PCDSS mice had more proliferating (Ki67^+^) colonic epithelial cells (Figure 1h,j), more goblet (PAS^+^) cells (Figure 1i,k), and higher mRNA levels of tight junction protein (Figure 1l,m) than the NCDSS mice. Collectively, these data suggest that cholecystectomy, after a relatively long follow-up, ameliorates DSS-induced colitis and accelerates the repairing process.

### Cholecystectomy increases secondary BAs and species involved in BAs metabolism in colitis mice

**Figure 2.**
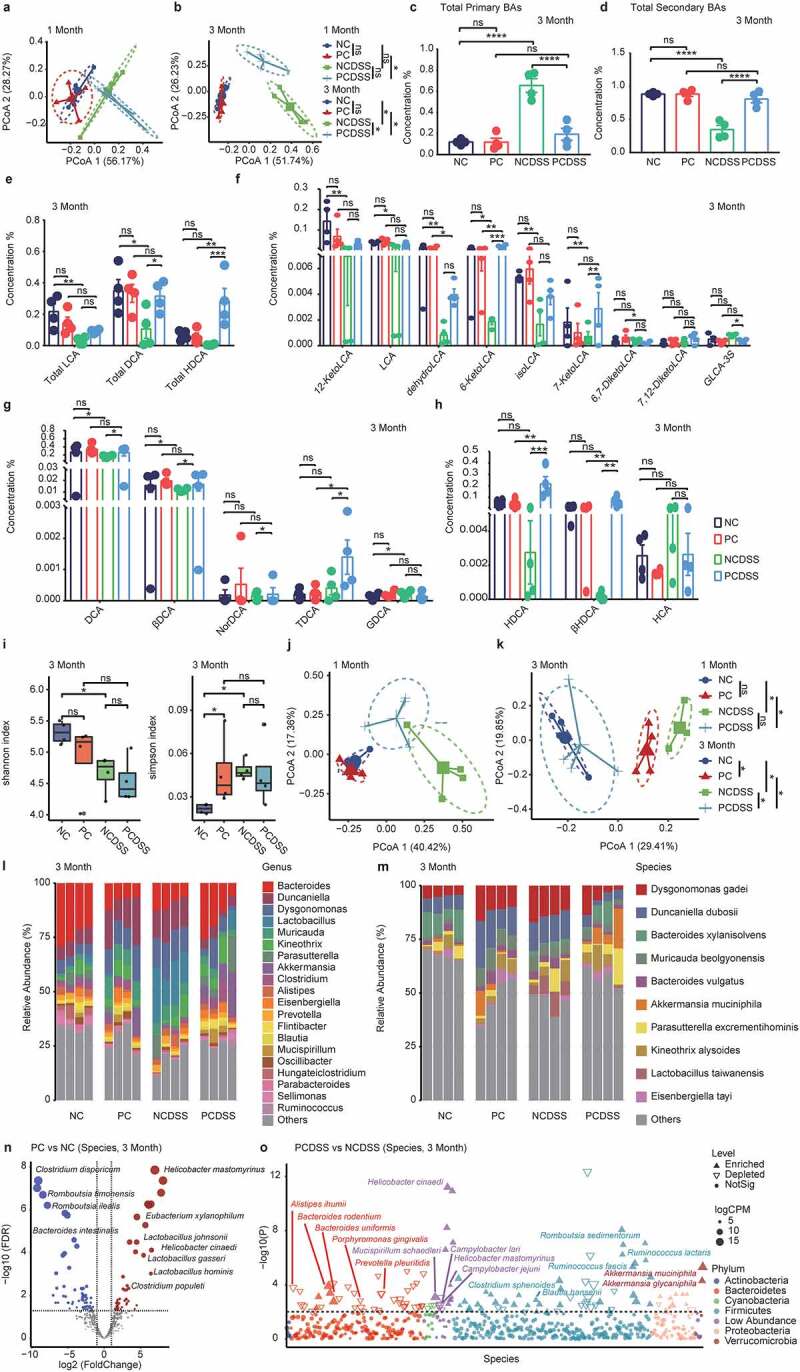
Cholecystectomy increases secondary BAs and species involved in BAs metabolism in colitis mice. a-b. PCoA analysis of fecal BAs profile on 1 month (a) or 3 months (b) after cholecystectomy. c-d. The relative concentration of fecal total primary BAs (c), total secondary BAs (d) 3^rd^ month. e. The relative concentration of fecal total lithocholic acid (LCA), total deoxycholic acid (DCA), and total hyodeoxycholic acid (HDCA) 3^rd^ month. f-h. The relative concentration of LCA(f), DCA(g), HDCA(h) and their derivatives. i. Shannon or Simpson index of fecal microbiota 3^rd^ month. j-k. Principal coordinate analysis (PCoA) of bacterial beta-diversity based on Bray Curtis distance on 1 month (j) or 3 months (k) after cholecystectomy. l-m. The bacterial composition at genus (l) or species (m) level at 3^rd^ month. n. Bacterial species with abundance differentiation between PC and NC mice in the volcano diagram at 3^rd^ month. o. Bacterial species with abundance differentiation between PCDSS and NCDSS mice in the Manhattan diagram at 3^rd^ month. Differences between the two groups were shown as point shape indicated OTU enriched, depleted, or not significant; point size indicated the abundance of OTU. Data are represented as mean ± SEM. *P < .05, **P < .01. ***P < .001, ****P < .0001, ns: not significant. DSS, dextran sulfate sodium; NC, normal control; NCDSS, normal control with DSS; PC, Cholecystectomy; PCDSS, Cholecystectomy with DSS. LCA, lithocholic acid; DCA, deoxycholic acid; HDCA, hyodeoxycholic acid.

After cholecystectomy, BAs were secreted into the intestinal tract continuously, and most studies showed increased secondary BAs concentrations in the PC patients consequently,^10–13^ so we tested fecal BAs in murine fecal samples. Although the PC mice showed similar fecal BA profiles with the NC mice after 3 months, we found increased secondary BAs levels in the PC mice after 3 months compared with pre-operative PC mice (Figure S1b-f). Principal coordinate analysis (PCoA) analysis showed the PCDSS and NCDSS mice had similar clusters at the first month after surgery (Figure 2a); while distinctly different clusters were shown at 3^rd^ month (Figure 2b). The fecal BAs profiles at the first month after cholecystectomy were displayed in Figure S2a-f. The PCDSS mice had higher fecal concentration of total secondary BAs and lower total primary BAs level than NCDSS mice at the 3^rd^ month (Figure 2c,d). As for secondary BAs, we found the PCDSS mice had increased total DCA, and total HDCA levels compared with the NCDSS mice at the third month (Figure 2e). Furthermore, the levels of LCA derivatives (6-ketoLCA, 7-ketoLCA, and GLCA-3S), DCA and its products (βDCA, NorDCA, and TDCA), HDCA and its derivative βHDCA significantly increased in the PCDSS mice at the 3^rd^ month (Figure 2f-h).

After BAs are secreted in the intestine, an array of bacteria gives rise to secondary bile acids through bio-transformations;^19^ fecal microbiota was also identified. The bacterial α diversity (Shannon and Simpson indices) was not altered after cholecystectomy under normal and inflammatory status (Figure 2i and Figure S2g, h). The PCoA analysis demonstrated significant differences at the 3^rd^ month (but not for the 1^st^ month) between the PC and NC mice, and between the PCDSS and NCDSS mice (Figure 2j,k). The compositional analysis showed the main bacteria in fecal bacteria at the phylum (Figure S2i, j), the genus (Figure 2l), and the species level (Figure 2m). We also figured out the differentiated bacterial contents between PC and NC mice at the 3^rd^ month at the species level (Figure 2n, 1^st^ month in Figure S2k). As a result, some species belonging to the secondary-BAs-producing genera^19^ (*Lactobacillus gasseri, Lactobacillus hominis, Lactobacillus johnsonii, Clostridium populeti*, and *Eubacterium xylanophilum*) and *Helicobacter* species were highly colonized at the gut lumen in the PC mice; inversely, lower potential pathogens occupied,^27–29^ such as *Romboutsia ilealis, Romboutsia timonensis, Clostridium disporicum*, and *Bacteroides intestinalis*. Additionally, compared with NCDSS mice, PCDSS mice also have a higher relative abundance of species belonging to secondary-BAs-producing genus^19^ (*Bacteroides rodentium, Bacteroides uniformis, Ruminococcus lactaris, Ruminococcus faecis*, and *Clostridium sphenoidese*), immuno-modulating species^30–32^ (including *Akkermansia glycaniphila, Akkermansia muciniphila, Romboutsia sedimentorum, Blautia hansenii* and *Mucispirillum schaedleri*) and *Helicobacter* species. In contrast, several potential pathogenic species^33–37^ including *Alistipes ihumii, Campylobacter jejuni, Campylobacter lari, Porphyromonas gingivalis*, and *Prevotella pleuritidis* were reduced in the PCDSS mice (Figure 2o, 1^st^ month in Figure S2l).

These results suggested that cholecystectomy induced a significant undulation of fecal microbiota and BAs profiles under colitis status, which featured a higher relative abundance of species involved in immuno-regulation and BAs metabolism, along with increased secondary BAs levels.

### Specific secondary BAs mitigate DSS-induced murine colitis

**Figure 3.**
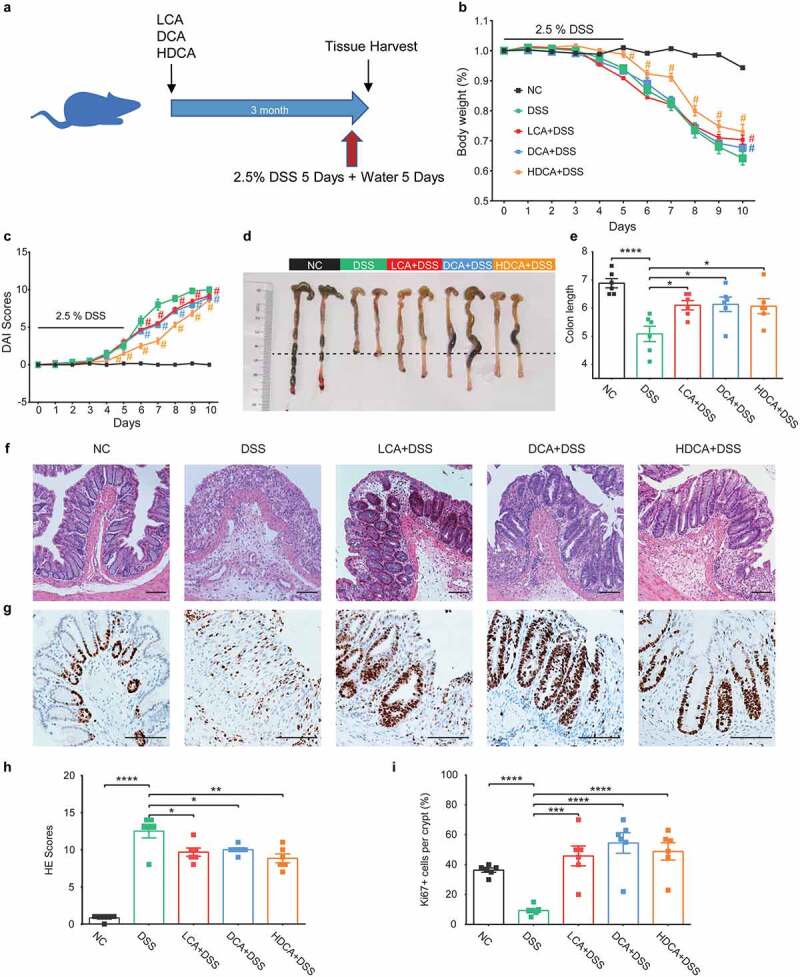
Secondary BAs also mitigate DSS-induced murine colitis. a. Schematic diagram showing the overall design and complete timeline. Mice were treated with LCA, DCA, or HDCA (2 mM of each) in drinking water for 3 months before inducing colitis. b. Body weight change (relative to starting weight, set as 100%) during the course of DSS-induced colitis. c. Disease activity index during colitis models. d-e. Representative colonic images and colon length. f. Representative hematoxylin and eosin-stained sections of the colon. g. Immunohistochemical staining of Ki67 in colon sections. h. histological scores of colons. i. Quantitative analysis of colonic ki67+ cells. Scale bar, 100 μm. Data are represented as mean ± SEM. N = 6–8 per group. *P < .05, **P < .01, ***P < .001, ****P < .0001, ^#^P < .05 compared with DSS. ns: not significant. BAs, bile acids; DCA, deoxycholic acid; DSS, dextran sulfate sodium; HDCA, hyodeoxycholic acid; HE, hematoxylin & eosin; LCA, lithocholic Acid; NC, normal control.

To investigate whether the secondary BAs, accumulated after cholecystectomy, could ameliorate colitis, the mice were treated with LCA, DCA, or HDCA in drinking water for three months before inducing colitis (Figure 3a). As DSS-induced colitis progressed, all secondary BAs groups reduced their colitis signs, as shown by body weight loss (Figure 3b), DAI scores (Figure 3c), and colonic length (Figure 3d,e). These secondary BAs also significantly reduced the inflammatory cells infiltration and histologic scores in the colon (Figure 3f,h). In keeping with the colonic proliferation status of the PCDSS mice, LCA, DCA, or HDCA treatment also displayed more proliferating (Ki67^+^) epithelial cells (Figure 3g,i) in murine colitis models. In brief, LCA, DCA, or HDCA, which accumulated after cholecystectomy, mitigated experimental colitis and accelerated mucosal repair.

### Cholecystectomy inhibits monocytes/macrophages recruitment to relieve colitis in mice

**Figure 4.**
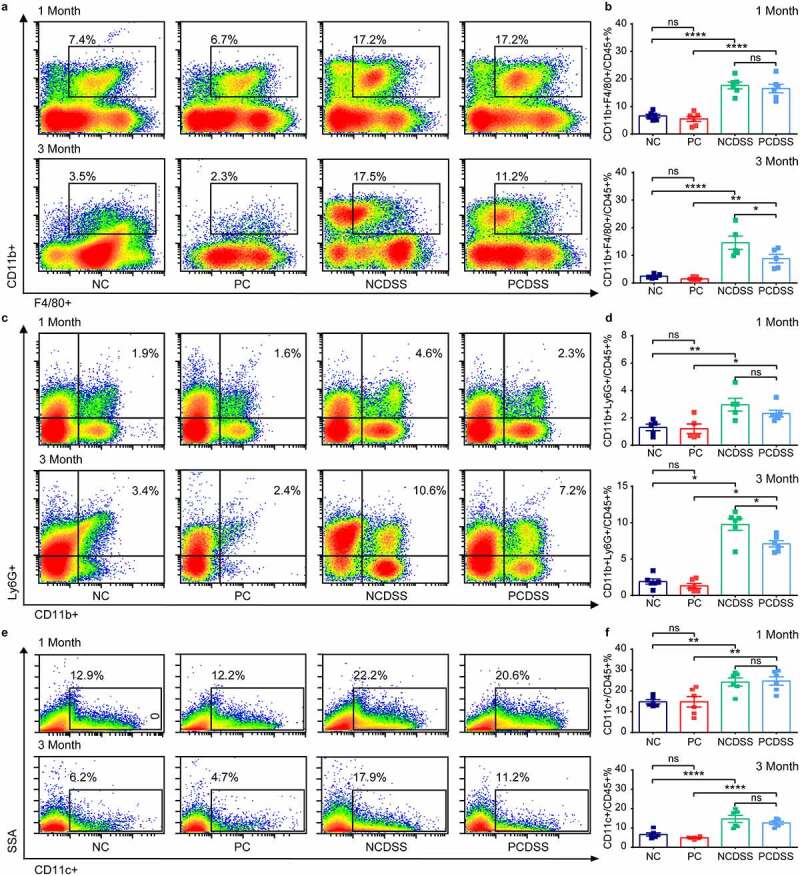
Cholecystectomy inhibits DSS-induced macrophages responses. Mice were induced colitis by DSS treatment at first or third month after cholecystectomy. a-b. Representative flow cytometric plots (a) and quantitative analysis (b) of the CD45+ F4/80+ CD11b+ colonic macrophages. c-d. Representative flow cytometric plots (c) and quantitative analysis (d) of the CD45+ CD11b+ Ly6G+ colonic Neutrophils. e-f. Representative flow cytometric plots (e) and quantitative analysis (f) of the CD45+ CD11c+ colonic dendritic cells. Data are represented as mean ± SEM. N = 5–6 per group. *P < .05, **P < .01, ***P < .001, ****P < .0001. ns: not significant. DSS, dextran sulfate sodium; NC, normal control; NCDSS, normal control with DSS; PC, Cholecystectomy; PCDSS, Cholecystectomy with DSS.

We next investigated which immune cell types were at work under inflammatory status. After isolating immunocytes from colonic tissues, flow cytometry was performed to evaluate immune cell subsets (gating strategies in Figure S3a, b). Per the mild changes in colitis signs, we failed to observe altered immune cells between the PCDSS and NCDSS mice at the first month (Figure 4a-f, Figure S4a-f). Notably, cholecystectomy significantly inhibited DSS-induced CD45^+^CD11b^+^F4/80^+^ macrophages (Figure 4a,b) and CD45^+^CD11b^+^Ly6G^+^ neutrophils (Figure 4c,d) responses at 3^rd^ month; while had little effects on CD45^+^CD11c^+^ dendritic cells (Figure 4e,f), CD45^+^CD3^+^CD4^+^ helper T lymphocytes (Figure S4a, b), CD45^+^CD3^+^CD8^+^ cytotoxic T lymphocytes (Figure S4c, d) and CD45^+^CD3^−^B220^+^ B cells (Figure S4e, f) in the colon.

**Figure 5.**
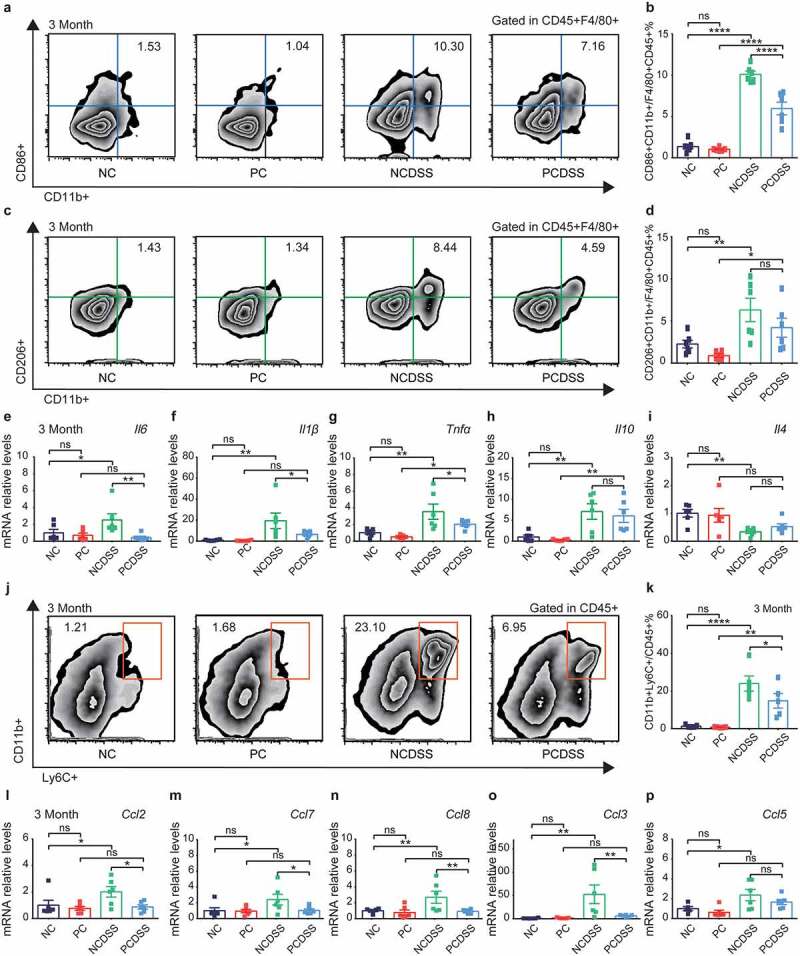
Cholecystectomy inhibits monocytes/macrophages recruitment to relieve colitis in mice. Mice were induced colitis by DSS treatment at third month after cholecystectomy. a-b. Representative flow cytometric plots (a) and quantitative analysis (b) of the colonic CD45+ F4/80+ CD11b+ CD86+ macrophages. c-d. Representative flow cytometric plots (c) and quantitative analysis (d) of the colonic CD45+ F4/80+ CD11b+ CD206+ macrophages. e-g. Relative mRNA expression of proinflammatory cytokines *Il6* (e), *Il1β* (f) and *Tnfα* (g) in colon tissues. h-i. Relative mRNA expression of anti-inflammatory cytokines *Il10* (h) and *Il4* (i) in colon tissues. j-k. Representative flow cytometric plots (j) and quantitative analysis (k) of the colonic CD45+ CD11b+ Ly6C+ monocytes. l-p. Relative mRNA expression of chemokines *Ccl2* (l), *Ccl7* (m), *Ccl8* (n), *Ccl3* (o) and *Ccl5* (p) in colon tissues. Data are represented as mean ± SEM. N = 5–6 per group. *P < .05, **P < .01, ***P < .001, ****P < .0001. ns: not significant. Ccl, chemokine (C-C motif) ligand; DSS, dextran sulfate sodium; Il, interleukin; NC, normal control; NCDSS, normal control with DSS; PC, Cholecystectomy; PCDSS, Cholecystectomy with DSS; Tnfα, tumor necrosis factor α.

Based on the above-mentioned results indicating the critical role of BAs in intestinal inflammation. Additionally, T and B lymphocytes express low levels of bile acid receptors (BARs), but modulation of monocytes, macrophages, and DC cells by BARs has been widely reported.^38^ We further chose macrophages and explored whether cholecystectomy affected macrophages’ differentiation to regulate immune responses. We found that the PCDSS mice had lower intestinal pro-inflammatory CD45^+^F4/80^+^CD11b^+^CD86^+^ macrophages than the NCDSS mice (Figure 5a,b); and the levels of anti-inflammatory CD45^+^F4/80^+^CD11b^+^CD206^+^ macrophages were similar between two groups in the colon (Figure 5c,d). Moreover, cholecystectomy also reduced mRNA levels of pro-inflammatory cytokines (*Il1β, Il6, Tnfα*, Figure 5e-g); while it did not affect anti-inflammatory cytokines (*Il4, Il10*, Figure 5h,i) in colonic tissues under colitis status. In summary, cholecystectomy reduced the total macrophages and pro-inflammatory macrophages subsets; but had little effect on anti-inflammatory macrophages in colitis models, implying that cholecystectomy might play a pivotal role in the origin of intestinal macrophages.

As most intestinal macrophages are from constant replenishment by circulating monocytes,^39^ we next explored whether cholecystectomy could regulate monocytes’ mobilization into the inflamed colon. Along with the reduced intestinal macrophage population, intestinal monocytes (CD45^+^CD11b^+^Ly6C^+^) were significantly lower in the PCDSS mice than in the NCDSS mice at 3^rd^ month (Figure 5j,k). The secretion of chemokines is required to support the recruitment of monocytes.^39^ The PCDSS mice also showed dramatic reductions in the colonic mRNA levels of monocytes-related chemokine (C–C motif) ligand 2 (*Ccl2), Ccl7, Ccl8*, and *Ccl3* relative to NCDSS mice (Figure 5l-p). In brief, cholecystectomy inhibited mRNA levels of chemokines, which were essential to mobilizing monocytes into the inflamed colon to supply macrophages.

### Specific secondary BAs regulate monocytes/macrophages mobilizations in mice

**Figure 6.**
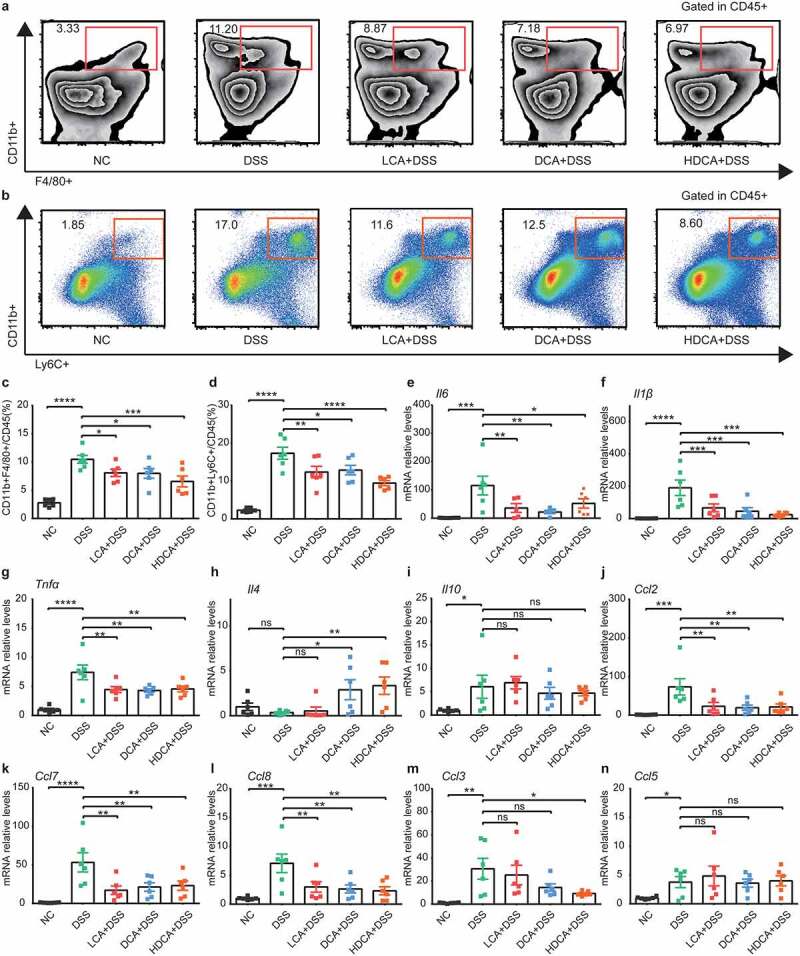
Secondary BAs also regulate monocytes/macrophages mobilization in mice. Mice were treated with LCA, DCA, or HDCA in drinking water for 3 months before inducing colitis. a-b. Representative flow cytometric plots of the colonic CD45+ F4/80+ CD11b+ macrophages (a) and CD45+ CD11b+Ly6C+ monocytes (b). c-d. Quantitative analysis of macrophages (c) and monocytes (d). e-g. Relative mRNA expression of proinflammatory cytokines *Il6* (e), *Il1β* (f), and *Tnfα* (g) in colon tissues. h-i. Relative mRNA expression of anti-inflammatory cytokines *Il4* (h) and *Il10* (i) in colon tissues. j-n. Relative mRNA expression of chemokines *Ccl2* (j), *Ccl7* (k), *Ccl8* (l), *Ccl3* (m) and *Ccl5* (n) in colon tissues. Data are represented as mean ± SEM. N = 6–8 per group. *P < .05, **P < .01, ***P < .001, ****P < .0001. ns: not significant. Ccl, chemokine (C-C motif) ligand; DCA, deoxycholic acid; DSS, dextran sulfate sodium; HDCA, hyodeoxycholic acid; *Il*, interleukin; LCA, lithocholic Acid; NC, normal control; PC, Cholecystectomy; Tnf, tumor necrosis factor α.

To directly confirm whether accumulated secondary BAs after cholecystectomy regulated colonic monocytes and macrophages, we also conducted flow cytometry in colitis models after secondary BAs supplements for three months. Secondary BAs (LCA, DCA, and HDCA) reproducibly reduced colonic monocytes and macrophages in colitis mice (Figure 6a-d). The secondary BAs consistently showed a significant reduction in the mRNA levels of pro-inflammatory cytokines (*Il6, Il1β, Tnfα*, Figure 6e-g) and some monocytes-related chemokines (*Ccl2, Ccl7, Ccl8*, Figure 6j-n) in colitis mice. As for anti-inflammatory cytokines, LCA, DCA, and HDCA did not alter the mRNA levels of *Il10* while DCA and HDCA increased the mRNA levels of *Il4* in colitis mice (Figure 6h,i). These results indicated that secondary BAs accumulated after cholecystectomy played a crucial role in regulating chemokines expression, followed by reduced recruitment of monocytes and replenishments of macrophages.

### Activation of Liver X receptor α by secondary BAs downregulates chemokines in THP-1 derived macrophages

The BAs are modified by microbes metabolically and also serve as signaling molecules, which activate BA receptors (BARs).^19^ Thus, we explored whether the BARs were affected in the aforementioned murine models. By comparing colonic mRNA levels of various BARs in colitis animals, most BARs levels were increased in PCDSS mice (Figure S5a), such as *Gpbar1*(TGR5), *Vdr, Nr1h3* (LXRα), *Nr1h2* (LXRβ), *Nr1i2* (PXR), and *Nr1i3* (CAR). Similarly, the secondary BAs treatments showed increased levels of some BARs, including *Nr1h4*(FXR), *Gpbar1, Nr1h3*, and *Nr1h2* (Figure S5b). The anti-inflammatory effects of FXR and GPBAR1 in macrophages have been widely reported.^40^ The LXRs are highly expressed in macrophages.^41^ To confirm whether the inhibitory action of secondary BAs is also mediated through LXRs, we also tested the LXR target gene (*Abca1, Abcg1*) and found the mRNA levels were also increased along with LXRs levels (Figure S5c). These results revealed that LXRs signaling might be involved in the process of regulating immune responses.

**Figure 7.**
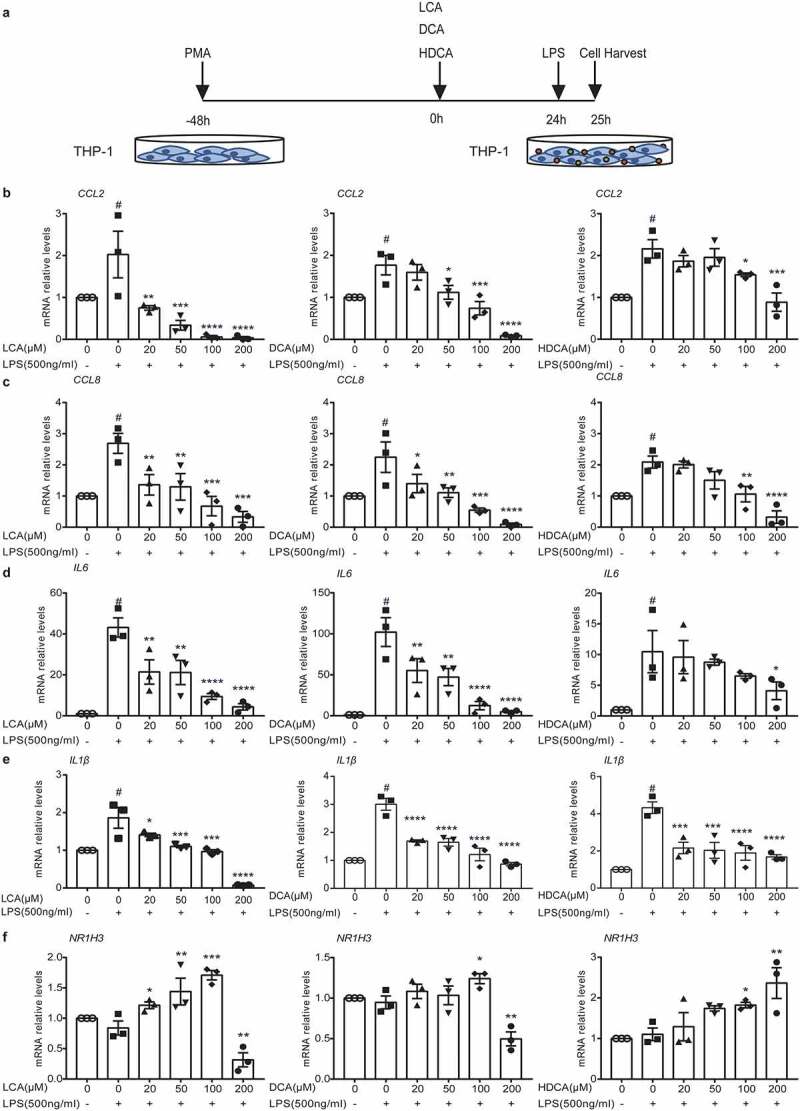
Secondary BAs downregulate inflammatory cytokine and chemokines in THP-1 derived macrophages. a. Schematic illustration. After incubation with 100 ng/ml PMA for 48 h to differentiate into macrophages, THP-1 derived macrophages were cultured with 20, 50, 100, and 200 μM secondary BAs (LCA, DCA or HDCA) for 24 h and then stimulated with 500 ng/ml LPS for 1 h. b-c. Relative mRNA expression of chemokines *CCL2* (b) and *CCL8* (c) in THP1 cells. d-e. Relative mRNA expression of proinflammatory cytokines *IL6* (d) and *IL1β* (e) in THP1 cells. f. Relative mRNA expression of bile acid receptor *NR1H3 (LXRα)* in THP1 cells. Data are represented as mean ± SEM. *P < .05, **P < .01, ***P < .001, ****P < .0001 compared with 0 μM BAs LPS+ group. #P < .05 compared with negative control. ns: not significant. CCL, chemokine (C-C motif) ligand; DCA, deoxycholic acid; HDCA, hyodeoxycholic acid; IL, interleukin; LCA, lithocholic Acid; LPS, Lipopolysaccharide; LXRα, Liver X receptor α; PMA, Phorbol-12-myristate-13-acetate.

**Figure 8.**
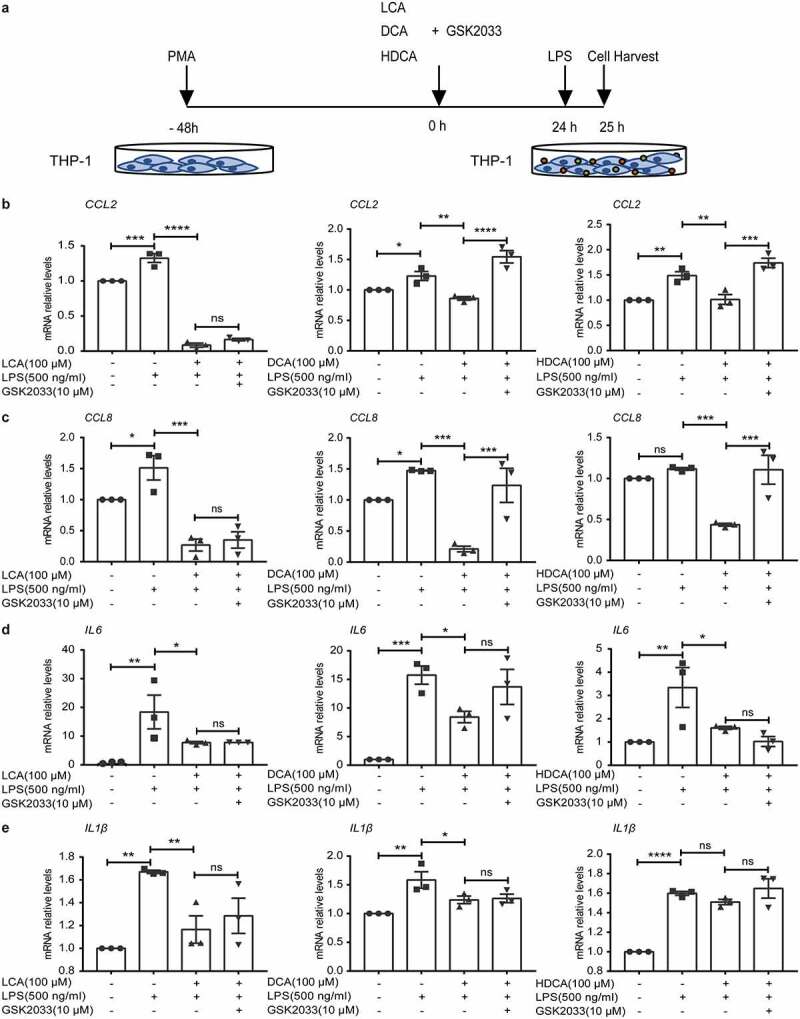
Activation of Liver X receptor by secondary BAs downregulates chemokines in THP-1 derived macrophages. a. Schematic illustration. After incubation with 100 ng/ml PMA for 48 h to differentiate into macrophages, THP-1 derived macrophages were pretreated with 100 μM secondary BAs (LCA, DCA or HDCA) and 10 μM an antagonist of LXR (GSK2033) for 24 h, and then stimulated with 500 ng/ml LPS for 1 h. b-c. Relative mRNA expression of proinflammatory cytokines *IL6* (b) and *IL1β* (c) in THP1 cells. d-e. Relative mRNA expression of chemokines *CCL2* (d) and *CCL8* (e) in THP1 cells. Data are represented as mean ± SEM. *P < .05, **P < .01, ***P < .001, ****P < .0001. ns: not significant. CCL, chemokine (C-C motif) ligand; DCA, deoxycholic acid; HDCA, hyodeoxycholic acid; IL, interleukin; LCA, lithocholic Acid; LPS, Lipopolysaccharide; PMA, Phorbol-12-myristate-13-acetate.

Based on the results mentioned above, secondary BAs inhibited inflammatory cytokines and monocytes chemoattractant proteins. We tried to identify the mechanism by which secondary BAs were at work *in vitro*. After differentiation into macrophages, THP-1 derived macrophages were cultured with 20, 50, 100, and 200 μM specific secondary BAs (LCA, DCA, or HDCA) for 24 h and then stimulated with LPS for one hour (Figure 7a). Data showed that the LCA, DCA, or HDCA inhibited mRNA levels of LPS-induced chemokines (*CCL2, CCL8*, Figure 7b,c) and inflammatory cytokines (*IL6, IL1β*, Figure 7d,e) responses in a dose-dependent manner. We also tested several BARs levels in THP-1 derived macrophages. As a result, there were dose-dependent increased effects of LCA, DCA, or HDCA on *Nr1h3* (LXRα) levels (Figure 7f), rather than other BARs (Figure S6a-d). Furthermore, the cells were pretreated with 100 μM secondary BAs and an LXR inhibitor GSK2033, then stimulated with LPS for one hour (Figure 8a). The inhibiting effects of DCA or HDCA on chemokines (*CCL2, CCL8*) were abrogated in the presence of LXR inhibitor (Figure 8b,c), but inflammatory cytokines (*IL6, IL1β*) were still suppressed (Figure 8d,e). The LXRα signaling seemed noncontributory in LCA-restrained chemokines secretions in macrophages (Figure 8b-e).

These results indicated that secondary BAs reduced LPS-induced chemokines expressions through activation of LXRα, following our *in vivo* findings that low chemokines levels after secondary BAs treatments in colitis models.

### Increased secondary BAs and related mucosal bacteria are negatively associated with peripheral monocytes in PC patients

**Figure 9.**
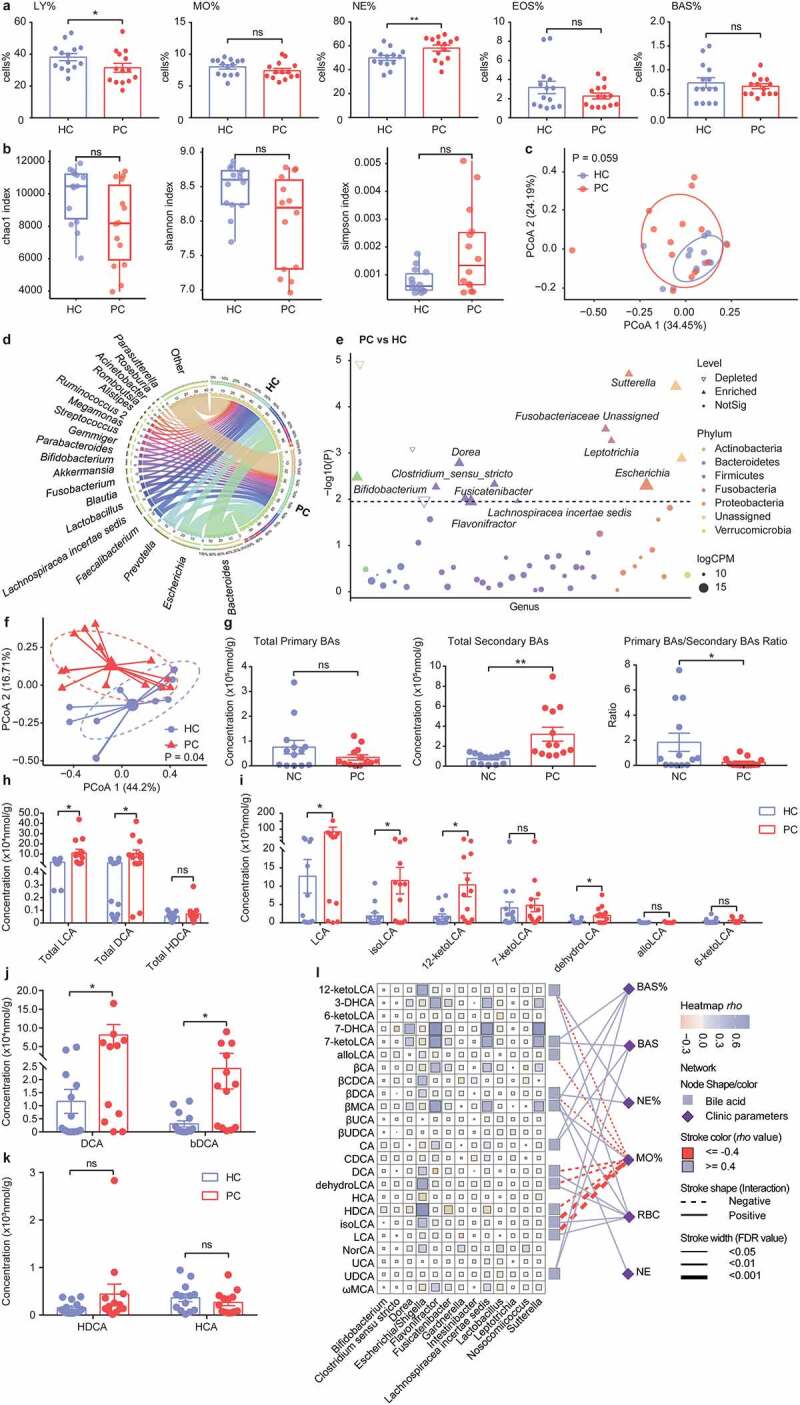
Mucosal microbiota and fecal BAs profile alterations after cholecystectomy. a. Proportions of LY, MO, NE, EOS, and BAS in peripheral blood. b. The alpha diversity of mucosal microbiota (Chao1 index, Shannon index, Simpson index). c. Principal coordinate analysis (PCoA) of bacterial beta-diversity based on Bray Curtis distance. d. Chordal graph to visualize the bacterial composition at the genus level. e. Bacterial species with abundance differentiation between PC and HC group in the Manhattan diagram. Differences between the two groups were shown as point shape indicated OTU enriched, depleted, or not significant; point size indicated the abundance of OTU. f. PCoA analysis of fecal BAs profile. g. The concentration of fecal total primary BAs, total secondary BAs, and primary BAs/secondary BAs ratio. h. The concentration of fecal total lithocholic acid (LCA), total deoxycholic acid (DCA), and total hyodeoxycholic acid (HDCA). i-k. The concentration of LCA, DCA, HDCA, and derivatives. l. Bacteria-BAs-immune cells correlation. Data are represented as mean ± SEM. *P < .05, **P < .01. ns: not significant. BAs, bile acids; BAS, basophilic granulocyte; CPM, counts per million; EOS, eosinophilic granulocyte; FC, fold change; HC, healthy controls; LY, lymphocyte; MO, monocytes; NE, neutrophil; PC, post-cholecystectomy.

To confirm whether the alterations of intestinal microbiota and BAs were associated with immune cells in the PC patients, we enrolled 14 PC patients and paired 14 healthy controls (HC) in this study (demographic, clinical, and endoscopic profiles were shown in Table 1 and Table S1). The PC patients had more gastrointestinal symptoms than HC, especially diarrhea (Table 1). There was a decreasing trend of peripheral lymphocytes, monocytes, eosinophils, and basophilic granulocytes while neutrophils were increased in the PC patients (Figure 9a).

**Table 1.** Baseline participant characteristics.

Characteristics	Healthy controls (*n = *14)	Post-cholecystectomy (*n = *14)	*p-*value
Sex, Men, No. (%)	6 (42.86%)	6(42.86%)	1.000
Age (years)	60.21 ± 2.35	59.43 ± 2.46	0.309
Height (m)	165.43 ± 2.57	165.50 ± 2.35	0.971
Weight (kg)	64.29 ± 3.61	69.71 ± 2.84	0.224
BMI (kg/m^2^)	23.23 ± 0.94	25.46 ± 0.92	0.109
Post-cholecystectomy duration(years)		9.14 ± 2.63	
Gastrointestinal Symptoms			
Total, No. (%)	4 (28.57%)	12 (85.71%)	0.006
Abdominal Distension, No. (%)	0 (0.00%)	3 (21.43%)	0.222
Diarrhea, No. (%)	0 (0.00%)	7 (50.00%)	0.006
Abdominal Pain, No. (%)	1 (7.14%)	4 (28.57%)	0.326
Acid Reflux, No. (%)	2 (14.29%)	3 (21.43%)	1.000
Constipation, No. (%)	1 (7.14%)	1 (7.14%)	1.000
Complications			
IBD, No. (%)	0 (0.00%)	0 (0.00%)	1.000
CRC, No. (%)	0 (0.00%)	1 (7.14%)	1.000
NAFLD, No. (%)	2 (14.29%)	7 (50.00%)	0.103
HBP, No. (%)	1 (7.14%)	6 (42.85%)	0.077
DM, No. (%)	0 (0.00%)	4 (28.57%)	0.098
HLP, No. (%)	1 (7.14%)	4 (28.57%)	0.326
CHD, No. (%)	0 (0.00%)	1 (7.14%)	1.000

Our published study showed significant changes of fecal bacteria in PC patients compared with HC subjects;^17^ we thus further tested mucosal microbiota in this study. The PC group had a lower Shannon and Chao1 index than the HC group without significance (Figure 9b). Then, PCoA analysis showed similar clusters between the PC and HC groups (Adonis *P* = .059, Figure 9c). The compositions of mucosal bacterial were quite different between the two groups at the genus level (Figure 9d). We evaluated differential abundance analysis using the Manhattan diagram (Figure 9e). Bacterial genera, such as *Bifidobacterium, Fusicatenibacter*, and *Sutterella*, were significantly enriched in the PC patients, often beneficial or inducing mild/negligible inflammatory responses.^42–46^ Notably, some harmful genera (*Leptotrichia, Dorea, Flavonifractor*, and Fusobacteriaceae Unassigned), which were reported to be linked with CRC,^47–49^ also increased in the PC patients.

We next conducted fecal BAs analysis, PCoA analysis displayed distinct clusters between the two groups (Figure 9f). Compared with the HC subjects, the PC patients had similar fecal concentrations of primary BAs and markedly increased secondary BAs levels, along with decreased primary/secondary BAs ratio (Figure 9g). As for secondary BAs, we found increased total LCA and total DCA concentrations in the PC patients compared with the HC subjects, while total HDCA levels were unaltered between the two groups (Figure 9h). Following the changes in total concentrations, the levels of LCA, DCA, and their derivatives significantly increased while the levels of HDCA and HCA were unchanged in the PC patients (Figure 9i-k). Additionally, we integrated data of fecal BAs, mucosal bacteria, and peripheral blood cells for conjoint analysis. The result showed that the proportion of peripheral monocytes was negatively associated with LCA and its derivatives (isoLCA, dehydroLCA, 12-ketoLCA, and alloLCA), DCA and its products (βDCA), HDCA (Figure 9l).

In this part, we found that the undulating profiles of gut microbiota and BAs in PC patients, especially increased secondary BAs and related bacterial contents, which were remarkably and negatively associated with the peripheral monocytes level, confirming that cholecystectomy might induce intestinal microenvironment changes to regulating monocytes/macrophages recruitment as mentioned in murine colitis models.

## Discussion

Until now, it is widely shown that PC patients have a higher risk of gastrointestinal complications, but no increased risk of IBD in PC patients has been reported in limited studies, and the mechanism was also not clearly underlined in animals.^4,5^ In our present time-course study, we have shown that cholecystectomy, after a relatively longer duration of post-operation, ameliorated DSS-induced colitis in mice, in keeping with our published research suggested that the follow-up period was a pivotal factor for clinical outcomes in PC patients.^17^ Based on decades-long duration in the PC patients, further studies are needed a markedly long follow-up year to clarify the role of duration after cholecystectomy in IBD patients.

Previous studies showed that primary and conjugated BAs were elevated, whereas secondary BAs were decreased substantially in IBD patients.^21,22^ However, we found that cholecystectomy restored normal secondary bile acid levels in DSS-treated mice at the 3^rd^ month. As previously described, PC patients also showed secondary BAs were elevated due to more exposure of BAs to microbiota.^10–13^ And the depletion of BA biotransformation and production capabilities in the microbiota of IBD patients was also implied.^23^ In our study, among increased species after cholecystectomy in murine colitis, *Bacteroides rodentium, Bacteroides uniformis, Ruminococcus lactaris, Ruminococcus faecis*, and *Clostridium sphenoidese* belong to the *Bacteroides, Clostridium*, and *Ruminococcus*, which involved in BAs deconjugation, 7-dehydroxylation, or epimerization.^40^ Some species with immuno-regulation were also increased. Mucus-penetrating *Akkermansia muciniphila* is a promising next-generation probiotic, which is negatively associated with metabolic syndromes and auto-immune diseases;^30^ *Mucispirillum schaedleri*, a mucolytic bacterium, has been reported to been partially modulated by pTreg cells;^31^ *Blautia hansenii* is classified into genus *Blautia*, which also has potential probiotic functions.^32^ As for reduced harmful bacteria, *Campylobacter jejuni* and *Campylobacter lari* are the leading pathogens of bacterial diarrheal diseases;^33,37^ *Porphyromonas gingivalis* is enriched in CRC patients and accelerates CRC development in mice;^34^ *Prevotella pleuritidis* has been linked to rheumatoid arthritis and gastric cancer.^35,36^ Different from the colitis status, bacterial alteration markedly changed while bile acids slightly altered in PC mice after 3 months under normal status, implying fecal microbiota might be significantly restructured to accommodate more frequent exposures of BAs after cholecystectomy. DSS treatment induced intestinal microbiota dysbiosis, which also includes BA-metabolizing bacteria, resulting in the more apparent alteration of bile acids under colitis status after cholecystectomy, which might relieve murine colitis.

Although the anti-inflammatory effects of secondary BAs were widely reported *in vitro*,^19^ their effects on colitis have been studied in recent years. As reported, LCA alleviated inflammation in murine colitis models.^24,50^ The role of DCA in regulating the immune system is controversial. DCA ameliorated DSS-induced colitis and exerted immuno-modulating effects *in vitro*;^24,51^ however, high-dose and long-term intake of DCA aggravated intestinal inflammation and accelerated the transition from intestinal adenoma to colonic adenocarcinoma.^25,26,52,53^ DCA has different effects depending on the specific conditions and combination of factors, which still require more studies in the future. HDCA has efficacy in treating animal models of metabolic disorders, such as hyperlipidemia, hypercholesterolemia, and type 2 diabetes mellitus.^54–57^ Recent studies reported that HDCA suppressed intestinal epithelial cell proliferation, accompanied by alteration of gut bacteria and BAs profiles, and the HDCA analogs protected against TNBS-induced colitis.^58,59^ We also confirmed specific secondary BAs (cholecystectomy-accumulated LCA, DCA, or HDCA) ameliorated DSS-induced colitis and accelerated mucosal repair.

Several mucosal immune cells (such as monocytes, macrophages, dendritic cells, and T cells) can be activated by BAs and exert their anti-inflammatory effects.^19^ Thus, we tested the intestinal immune responses in colitis models and found the intestinal macrophages response was reduced after cholecystectomy. Cholecystectomy also inhibited the elevation of pro-inflammatory macrophages rather than anti-inflammatory subsets under colitis status, implying that PC might affect the origin of intestinal macrophages. The intestinal macrophages mostly require constant replenishment by circulating monocytes throughout adulthood.^60^ During intestinal inflammation, inflammatory monocytes are recruited into inflamed tissues, depending on the chemokines (such as CCL2, CCL8, CCL7), and differentiate to mature intestinal macrophages.^39,60^ The PCDSS mice showed dramatic intestinal monocytes and chemokines reductions in this study. These data suggested that cholecystectomy modified the local environment (inhibiting chemokines secretions) to reduce monocyte recruitment, followed by decreased colonic macrophages. Furthermore, Cholecystectomy-accumulated secondary BAs inhibited chemokines expression, accompanied by reduced monocytes and macrophages under intestinal inflammation, revealing the critical role of secondary BAs in the immune-regulatory process.

BAs also severed as signaling molecules by activating several BARs.^19^ This study also found that LCA, DCA, or HDCA inhibited LPS-induced chemokines and inflammatory cytokine responses and increased LXRα levels in THP-1 derived macrophages. LCA and DCA have been shown to suppress inflammatory cytokines and chemokines from macrophages through activation of BARs (FXR, GPBAR1, and VDR) *in vitro*.^40^ Whether the inhibitory action of secondary BAs is also mediated through LXRs, which are highly expressed in macrophages,^41^ has not been elucidated. In addition, the immunoregulation of macrophages through LXRs has also been implicated. LXRs activation inhibits a set of inflammatory genes in activated macrophages.^61^ Our results revealed that DCA and HDCA (but not LCA) inhibited LPS-induced chemokines expressions through LXRα signaling. The LXRα signaling seems noncontributory in LCA-restrained chemokines secretions.^19,21,22^ Bile acids act as ligands, which affinity is different for bile acid-activated receptors.^38^ Natural bile acid agonists for FXR are CDCA > CA > LCA > DCA CDCA and GPBAR1 is mainly activated by secondary bile acids (LCA > DCA > CDCA > UDCA > CA).^40^ HDCA has also been shown as a natural ligand for LXRs and GPBAR1,^62^ but the affinity of LCA and DCA for LXRs is unknown, which still warrants further studies.

Our clinical results showed that PC patients have significantly increased secondary BAs (LCA, DCA), in consistent with published studies.^10–13^ Previous studies and our published results showed significant changes of fecal bacteria in PC patients,^14–17^ but mucosal microbiota alterations have not yet been reported. In this study, mucosal microbiota varied mildly after cholecystectomy, suggesting that the microbiota, sharped by BAs, could not act directly on the intestinal mucosa. Additionally, most of the secondary BAs-producing bacteria, mainly belonging to the obligate anaerobic Bacteroidetes and Firmicutes phyla, are involved primarily in fecal microbiota,^21,63^ which may be the reason for the slight changes in mucosal microbiota. Following the experiments in mice, we also found that the accumulation of secondary BAs was negatively associated with peripheral monocytes levels in the PC patients. These results suggested that cholecystectomy-induced secondary BAs might reduce monocyte recruitment, which still need further investigation in IBD patients with a history of cholecystectomy.

It has always been debated whether removing the gallbladder has long-term effects on human bodies. In this study, we found the immune regulatory effect of cholecystectomy on IBD through secondary BAs accumulation. Interestingly, a recent study indicated that cholecystectomy could partially alleviate long-term diabetes-induced gut microbiota dysbiosis.^64^ However, these results don’t mean cholecystectomy is harmless. It has been reported that cholecystectomy can increase the risk of CRC.^1^ Furthermore, the history of cholecystectomy was associated with a higher risk for incident colonic dysplasia in Crohn’s disease patients.^65^ Cholecystectomy’s potential carcinogenic effect still needs comprehensive consideration in further investigation.

**Figure 10.**
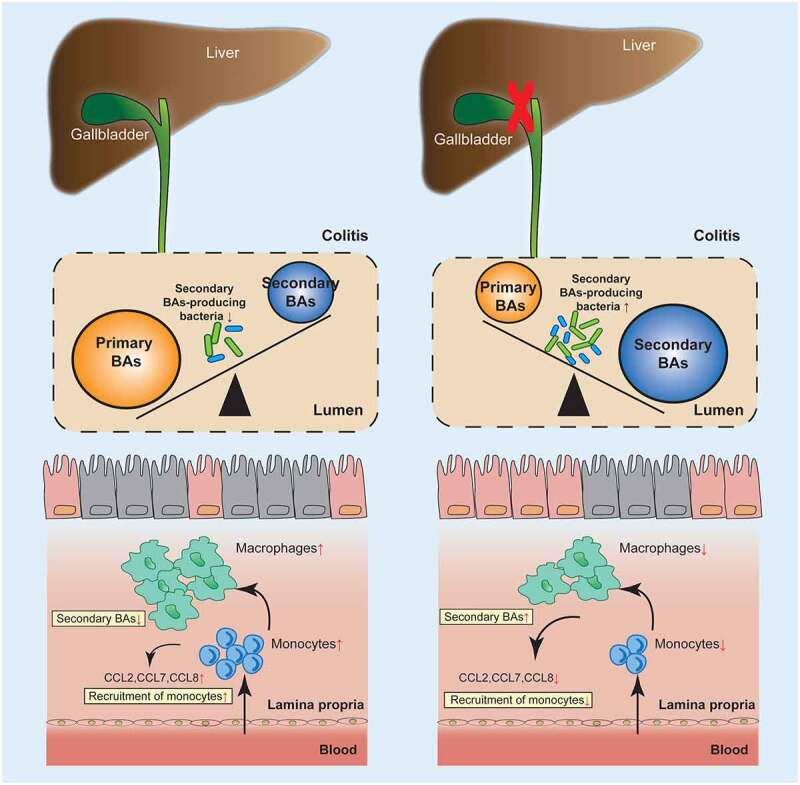
Graphic abstract. Cholecystectomy ameliorated colitis, along with increased secondary BAs levels and species involved in BAs metabolism. Cholecystectomy induced secondary BAs accumulation inhibited monocyte/macrophage recruitment, which might be mediated by the LXRα-related signaling pathway.

In conclusion, cholecystectomy-induced secondary BAs accumulation ameliorated colitis through inhibiting monocyte/macrophage recruitment, which might be mediated by the LXRα-related signaling pathway (Figure 10). Our findings showed the immune regulatory role of cholecystectomy in murine colitis after a relatively longer duration. The underlying mechanism preliminarily throw light on the epidemiological results that PC patients usually have gastrointestinal symptoms but no increased risk for IBD, which warrants further study.

## Methods and materials

### Animal experiments

Adult male C57BL/6 J mice (6–8 weeks) were purchased from the Vital River facility (Beijing, China). All the mice were housed in specific pathogen-free animal facilities with a 12 h-light/dark cycle. The animal use and humane care were approved by the Institutional Medical Ethics Review Board of Peking University People’s Hospital (No.2021PHE023).

The mice were allowed to acclimate for one week and divided into a cholecystectomy group (PC) and a negative control group (NC). After bile emptying of the filled gall bladder, the cystic duct was ligated and the gall bladder was removed in the PC group. The NC mice were received sham operations. One month or three months after the operation, the mice have induced colitis as described below.

Some groups of the mice were also treated with various BAs–containing water or regular water (changed every three days) for three months. The BAs tested were hyodeoxycholic acid (HDCA), DCA, LCA (2 mM of a single BA, Sigma-Aldrich). The mice have next induced colitis as below.

Murine colitis models were induced with 2.5% DSS (changed every 3 days, MP Biomedicals) in drinking water for 5 days and then switched to regular water or BAs–containing water for another 5 days. The mice were weighed and evaluated daily with DAI scores throughout experimental colitis. After the mice were sacrificed at specific time points, distal colonic tissue and feces were obtained for further analysis.

### Study subjects and sample collection

All subjects were enrolled from Peking University People’s Hospital from January 2018 to October 2018. Post-cholecystectomy patients (PC), who received operations above six months, were recruited. Healthy controls (HC) without biliary diseases, tumors, and traumatic ruptures were enrolled to match with PC patients (age and sex). Before sampling, all subjects were asked to avoid using probiotics and antibiotics for at least two weeks. Subsequently, all subjects were collected the colonic tissues for 16S rRNA sequencing and fecal samples for BAs profiles analysis. This clinical study was approved by the Institutional Medical Ethics Review Board of Peking University People’s Hospital (No. 2018PHB035-01). The written informed consent was obtained from all participants.

### Lamina propria immunocytes isolation

As described previously, the lamina propria immunocytes were isolated from colonic tissues.^66^ Briefly, colonic tissues were dissected and discarded fatty portions. Then colonic tissues were predigested in Hank’s Balanced Salt Solution (HBSS) containing 5 mM EDTA, 20 mM HEPES, 1 mM DTT, and 2% (vol/vol) FBS for 30 min at 37°C (all from Thermo Fisher Scientific). After discarding epithelium-containing supernatants and washing in PBS, the remaining tissues were minced into small pieces and digested with 0.2 mg/mL collagenase IV (Sigma-Aldrich), 0.2 mg/mL DNase I (Roche), 2% (vol/vol) FBS for 30 min at 37°C. The digested tissues were filtered through a 70-µm strainer, and the solutions were centrifuged at 500 g for 10 min to collect cells. Finally, the pellets were resuspended, and the lymphocytes were isolated by Percoll gradient centrifugation.

### Flow cytometry analysis

Lamina propria lymphocytes were stained with antibodies for 30 min at 4°C. The following anti-mouse antibodies (all from Biolegend) were used: Alexa Fluor 700-CD45 (Clone 30-F11), APC/Cyanine7-F4/80 (BM8), PerCP-CD11b (M1/70), Brilliant Violet 570-Ly6G (1A8), Brilliant Violet 421-CD86 (GL-1), FITC-CD206 (C068C2), PE-Ly6C (HK1.4), PerCP/Cyanine5.5-CD3 (17A2), BV421-CD4 (GK1.5), PerCP-CD8a (53–6.7) and FITC-B220 (RA3-6B2). All data were acquired on the Gallios instrument (Beckman) and analyzed using FlowJo X (TreeStar) or Beckman analysis software.

### HE staining and immunohistochemical staining (IHC)

Colon tissues were fixed by 4% paraformaldehyde, embedded in paraffin, and cut into 4-μm-thick sections. The sections were stained with standard hematoxylin and eosin (H&E) staining and independently scored by two double-blinded investigators. After deparaffinization and rehydration, sections were blocked with 3% hydrogen peroxide. After antigen retrieval, the colon tissue slides were incubated with the primary and secondary antibodies, then 3,3′-diaminobenzidine and hematoxylin were used. The sections were observed under an optical microscope and independently counted by two double-blinded investigators.

### Cell experiments

THP-1 cells, a human monocytic cell line, were cultured in RPMI 1640 medium containing 10% FBS and 1% penicillin–streptomycin (all from Thermo Fisher Scientific). For differentiation into macrophages, cells were incubated with 100 ng/ml Phorbol 12-myristate 13-acetate (PMA) (Biolegend) for 48 h. After exchanging medium, cells were cultured with 20, 50, 100, and 200 μM specific BAs (LCA, DCA, or HDCA, Sigma-Aldrich) for 24 h, then stimulated with 500 ng/ml LPS (Sigma-Aldrich) for 1 h. For macrophage BARs signaling assays, cells were pretreated with 100 μM BAs and 10 μM an antagonist of LXR (GSK2033, Sigma-Aldrich) for 24 h, and then stimulated with 500 ng/ml LPS for 1 h.

### RNA extraction and real-time quantitative PCR

Total RNA from colon tissues or macrophages was isolated using TRIzol reagent (Thermo Fisher Scientific). Then, a reverse transcriptase kit (Thermo Fisher Scientific, USA) was used to generate cDNA. qRT-PCR was conducted on the Applied Biosystems StepOne Plus Real-Time PCR System with SYBR Green Master Mix (Thermo Fisher Scientific). The primer sequences are displayed in Supporting Information Table S2.

### BAs detection

All of the BA standards were obtained from Steraloids and TRC Chemicals or synthesized in the Metabo-Profile laboratory (China). Ten stable isotope-labeled standards were obtained from C/D/N Isotopes (Canada) and Steraloids (USA). Internal Standard (IS) concentrations were kept constant at all the calibration points. Ultra-Performance liquid chromatography coupled to tandem mass spectrometry (UPLC-MS/MS) system (ACQUITY UPLC-Xevo TQ-S, Waters) was used to quantitate BAs in this project. The raw data files generated by UPLC-MS/MS were processed using the QuanMET software (Metabo-Profile) to perform peak integration, calibration, and quantitation for each metabolite. SIMCA 14.1 (32 bit) (Umetrics) was used for data analysis.

### 16S rRNA bacterial microbiota analysis

Mucosal samples from PC patients were collected for Illumina Hiseq 2000 sequencing. The bacterial 16S rRNA V3–V4 region was amplified using paired primers (357 F/806 R). The Illumina reads were sorted into different samples according to their barcoded index sequences. Both Vsearch v2.8.1 and Usearch v10 bit 32 were used in sequencing analysis. Two-side reads were merged, low low-frequency reads were removed, and high-quality reads were used for the subsequent analysis. The amplicon sequence variants (ASVs) method was performed to filter chimeras with Unoise3. ASVs were aligned using the Vsearch and taxonomically classified with the reference sequence rdp_16s_v16_sp.fa. Subsequently, the mice feces were also accumulated for bacterial profiling; while to profile bacterial contents at a resolution of the species, the Oxford Nanopore sequencing was performed with the MinION MK1B device. The raw sequencing data were analyzed with the MinKNOW platform, and reads were annotated with the NCBI 16S database. The sequences generated in the present study are available through the NCBI Sequence Read Archive (accession number).

### Statistical analysis and data visualization

Statistical analysis was performed using GraphPad Prism 9 (GraphPad Software) or SPSS 25.0 (IBM). Continuous variables were displayed as mean and standard error. Paired clinical studies were analyzed by Paired *t*-test or Wilcoxon matched-pairs signed-rank test. Differences between mice groups were evaluated by one-way analysis of variance (ANOVA) followed by Fisher’s LSD test or Student’s *t*-test. A *P* value ≤ .05 was defined as statistically significant.

## Supplementary Material

Supplemental Material

## Data Availability

The data that support the findings of this study have been deposited in the Genome Sequence Archive of the National Genomics Data Center, Beijing Institute of Genomics (China National Center for Bioinformation), and Chinese Academy of Sciences under accession number CRA006293, CRA006305 and are publicly accessible at: https://bigd.big.ac.cn/gsa. All data are available, further inquiries can be directed to the corresponding author.
