# Supplementary material for: Cholecystectomy-induced secondary bile acids accumulation ameliorates colitis through inhibiting monocyte/macrophage recruitment
Source: Gut Microbes. 2022 Sep 1;14(1):2107387. doi: 10.1080/19490976.2022.2107387 (PMC9450905; doi:10.1080/19490976.2022.2107387)
Supplement: Supplemental Material [file KGMI_A_2107387_SM2985.zip › Supplementary material.docx]

## Table S1. Characteristics of Endoscopic Findings.

| Characteristics | Healthy controls  (*n=*14) | Post-cholecystectomy  (*n=*14) | *p-*value |
| --- | --- | --- | --- |
| **Colonoscope** |  |  |  |
| colorectal polyps, No. (%) | 11 (78.57%) | 10 (71.43%) | 1.000 |
| Polyp size ≥6 mm, No. (%) | 2 (14.29%) | 3 (21.43%) | 1.000 |
| Polyp number≥3, No. (%) | 2 (14.29%) | 2 (14.29%) | 1.000 |
| Polyp location |  |  |  |
| Ileal, No. (%) | 1 (7.14%) | 1 (7.14%) | 1.000 |
| Colonic, No. (%) | 8 (57.14%) | 8 (57.14%) | 1.000 |
| Rectal, No. (%) | 3 (21.43%) | 4 (28.57%) | 1.000 |
| Pathologic manifestations |  |  |  |
| Inflammation, No. (%) | 5 (35.71%) | 2 (14.29%) | 0.385 |
| Tubular adenoma, No. (%) | 6 (42.86%) | 6 (42.86%) | 1.000 |
| Low-grade dysplasia, No. (%) | 5 (35.71%) | 4 (28.57%) | 1.000 |
| High-grade dysplasia, No. (%) | 0 (0.00%) | 1 (7.14%) | 1.000 |
| **Fecal occult blood** | 0 (0.00%) | 4 (28.57%) | 0.098 |
| **Gastroscope** |  |  |  |
| CAG, No. (%) | 6 (42.86%) | 4 (28.57%) | 0.695 |
| GERD, No. (%) | 2 (14.29%) | 5 (35.71%) | 0.385 |
| BR, No. (%) | 1 (7.14%) | 2 (14.29%) | 1.000 |
| GP, No. (%) | 1 (7.14%) | 3 (21.43%) | 0.596 |
| Pathologic manifestations |  |  |  |
| Intestinal Metaplasia, No. (%) | 6 (42.86%) | 4 (28.57%) | 0.695 |
| Atrophy, No. (%) | 2 (14.29%) | 2 (14.29%) | 1.000 |
| Dysplasia, No. (%) | 1 (7.14%) | 0 (0.00%) | 1.000 |
| HP, No. (%) | 1 (7.14%) | 2 (14.29%) | 1.000 |

## Table S2. Primers.

| Species | Genes | Forward(5’-3’) | Reverse(5’-3’) |
| --- | --- | --- | --- |
| Mouse | Il6 | TAGTCCTTCCTACCCCAATTTCC | TTGGTCCTTAGCCACTCCTTC |
| Mouse | Il1b | GCAACTGTTCCTGAACTCAACT | ATCTTTTGGGGTCCGTCAACT |
| Mouse | Tnf | CAGGCGGTGCCTATGTCTC | CGATCACCCCGAAGTTCAGTAG |
| Mouse | il4 | GGTCTCAACCCCCAGCTAGT | GCCGATGATCTCTCTCAAGTGAT |
| Mouse | il10 | GCTGGACAACATACTGCTAACC | ATTTCCGATAAGGCTTGGCAA |
| Mouse | Muc2 | CCGACTTCAACCCAAGTGAT | GAGCAAGGGACTCTGGTCTG |
| Mouse | Ccl2 | TAAAAACCTGGATCGGAACCAAA | GCATTAGCTTCAGATTTACGGGT |
| Mouse | Ccl7 | CCACATGCTGCTATGTCAAGA | ACACCGACTACTGGTGATCCT |
| Mouse | Ccl8 | TCTACGCAGTGCTTCTTTGCC | AAGGGGGATCTTCAGCTTTAGTA |
| Mouse | Ccl3 | TGTACCATGACACTCTGCAAC | CAACGATGAATTGGCGTGGAA |
| Mouse | Ccl5 | TTTGCCTACCTCTCCCTCG | CGACTGCAAGATTGGAGCACT |
| Mouse | Tjp1 | GCCGCTAAGAGCACAGCAA | GCCCTCCTTTTAACACATCAGA |
| Mouse | Ocln | TGAAAGTCCACCTCCTTACAGA | CCGGATAAAAAGAGTACGCTGG |
| Mouse | Nr1h2 | GCCTGGGAATGGTTCTCCTC | AGATGACCACGATGTAGGCAG |
| Mouse | Nr1h3 | CTGATTCTGCAACGGAGTTGT | GACGAAGCTCTGTCGGCTC |
| Mouse | Nr1h4 | GGCAGAATCTGGATTTGGAATCG | GCCCAGGTTGGAATAGTAAGACG |
| Mouse | Nr1i3 | GGGCCACAGGCTATCATTTCC | CCTCATGCCAACATTTAGACACT |
| Mouse | Nr1i2 | GATGGAGGTCTTCAAATCTGCC | GGCCCTTCTGAAAAACCCCT |
| Mouse | Vdr | GAATGTGCCTCGGATCTGTGG | ATGCGGCAATCTCCATTGAAG |
| Mouse | Gpbar1 | TGCTTCTTCCTAAGCCTACTACT | CTGATGGTTCCGGCTCCATAG |
| Mouse | Gapdh | AGGTCGGTGTGAACGGATTTG | TGTAGACCATGTAGTTGAGGTCA |
| Human | IL6 | ACTCACCTCTTCAGAACGAATTG | CCATCTTTGGAAGGTTCAGGTTG |
| Human | IL1B | ATGATGGCTTATTACAGTGGCAA | GTCGGAGATTCGTAGCTGGA |
| Human | TNFA | CCTCTCTCTAATCAGCCCTCTG | GAGGACCTGGGAGTAGATGAG |
| Human | CCL2 | CAGCCAGATGCAATCAATGCC | TGGAATCCTGAACCCACTTCT |
| Human | CCL8 | TGGAGAGCTACACAAGAATCACC | TGGTCCAGATGCTTCATGGAA |
| Human | NR1H2 | AGAAGATTCGGAAACAACAGCA | GCTGGATCATTAGTTCTTGAGCC |
| Human | NR1H3 | CCTTCAGAACCCACAGAGATCC | ACGCTGCATAGCTCGTTCC |
| Human | NR1H4 | AACCATACTCGCAATACAGCAA | ACAGCTCATCCCCTTTGATCC |
| Human | NR1I3 | GATGCTGGCATGAGGAAAGAC | TTGCTCCTTACTCAGTTGCAC |
| Human | NR1I2 | AAGCCCAGTGTCAACGCAG | GGGTCTTCCGGGTGATCTC |
| Human | VDR | GTGGACATCGGCATGATGAAG | GGTCGTAGGTCTTATGGTGGG |
| Human | GPBAR1 | CCCAGGCTATCTTCCCAGC | GCCAGGACTGAGAGGAGCA |
| Human | GAPDH | GGAGCGAGATCCCTCCAAAAT | GGCTGTTGTCATACTTCTCATGG |

Figure S1. BAs profiles in PC mice at 3^rd^ month compared with pre-operation mice.

a. Body weight change (weekly, relative to starting weight, set as 100%) during the course after cholecystectomy. b.The proportion of fecal total primary BAs (c), total secondary BAs. c.The proportion of fecal total lithocholic acid (LCA), total deoxycholic acid (DCA), and total hyodeoxycholic acid (HDCA). (d-f) The proportion of LCA(d), DCA(e), HDCA(f), and their derivatives. *P < 0.05, **P < 0.01. ns: not significant. PC-Before, Mice before receiving cholecystectomy; PC-After2M, Mice after receiving cholecystectomy for 3 months; BAs, bile acids; DCA, deoxycholic acid; HDCA, hyodeoxycholic acid; il, interleukin; LCA, lithocholic Acid;

Figure S2. Cholecystectomy alters fecal microbiota and BAs profiles in colitis mice. a-b. The concentration of fecal total primary BAs (a), total secondary BAs (b) on 1 month after cholecystectomy. c. The concentration of fecal total lithocholic acid (LCA), total deoxycholic acid (DCA), and total hyodeoxycholic acid (HDCA) on 1 month after cholecystectomy. d-f. The concentration of LCA(d), DCA(e), HDCA(f), and their derivatives. g-h. Shannon index (g) or Simpson index (h) of fecal microbiota on 1 month after cholecystectomy. i-j. The bacterial composition at phylum level on 1 month or 3 months after cholecystectomy. k. Bacterial genus with abundance differentiation between PC and NC mice on 1 month after cholecystectomy in the volcano diagram. l. Bacterial species with abundance differentiation between PCDSS and NCDSS mice on 1 month after cholecystectomy in the Manhattan diagram. Differences between the two groups were shown as point shape indicated OTU enriched, depleted, or not significant; point size indicated the abundance of OTU. Data are represented as mean±SEM. *P < 0.05, ns: not significant. DSS, dextran sulfate sodium; NC, normal control; PC, Cholecystectomy; NCDSS, normal control with DSS; PCDSS, Cholecystectomy with DSS. LCA, lithocholic acid; DCA, deoxycholic acid; HDCA, hyodeoxycholic acid.

Figure S3. Flow cytometry gating strategies. a. Flow cytometry gating strategies for colonic macrophages, neutrophils, dendritic cells, and monocytes. Representative flow cytometric plots. b. Flow cytometry gating strategies for colonic lymphocytes. Representative flow cytometric plots.

Figure S4. Cholecystectomy has little effect on DSS-induced lymphocytes response. Mice were induced colitis by DSS treatment on the first or third month after cholecystectomy. a-b. Representative flow cytometric plots (a) and quantitative analysis (b) of the colonic helper T lymphocytes (CD45+CD3+CD4+). c-d. Representative flow cytometric plots (c) and quantitative analysis (d) of the colonic cytotoxic T lymphocytes (CD45+CD3+CD8+). e-f. Representative flow cytometric plots (e) and quantitative analysis (f) of the colonic B cells (CD45+CD3-B220+). Data are represented as mean±SEM. N=5-6 per group. *P < 0.05, **P < 0.01. ns: not significant. DSS, dextran sulfate sodium; NC, normal control; NCDSS, normal control with DSS; PC, Cholecystectomy; PCDSS, Cholecystectomy with DSS.

Figure S5. The levels of bile acid receptors in murine colon. a. Mice were induced colitis by DSS treatment on the third month after cholecystectomy. Relative mRNA expression of bile acid receptors in colon tissues. b. Mice were treated with LCA, DCA, or HDCA in drinking water for 3 months before inducing colitis. Relative mRNA expression of bile acid receptors in colon tissues. c. Relative mRNA expression of LXR target gene in colon tissues. Data are represented as mean±SEM. N=6 per group. *P < 0.05, **P < 0.01, ***P < 0.001, ****P < 0.0001. ns: not significant. Abca1, ATP binding cassette subfamily A member 1; Abcg1, ATP binding cassette subfamily G member 1; DSS, dextran sulfate sodium; NC, normal control; NCDSS, normal control with DSS; PC, Cholecystectomy; PCDSS, Cholecystectomy with DSS; LCA, lithocholic Acid; DCA, deoxycholic acid; HDCA, hyodeoxycholic acid; Nr1h2, nuclear receptor subfamily 1 group H member 2; Nr1h3, nuclear receptor subfamily 1 group H member 2; Nr1h4, nuclear receptor subfamily 1 group H member 4; Gpbar1, G protein-coupled bile acid receptor 1; Vdr, vitamin D receptor; Nr1i2, nuclear receptor subfamily 1 group I member 2; Nr1i3, nuclear receptor subfamily 1 group I member 3.

Figure S6. Secondary BAs affects inflammatory cytokines and bile acid receptors in THP-1 derived macrophages. Relative mRNA expression of bile acid receptor NR1H4(a), GPBAR1(b), VDR(c) and NR1H2(d) in THP1 cells. Data are represented as mean±SEM. #P< 0.05 compared with negative control. *P < 0.05; **P < 0.01; ***P < 0.001; ****P < 0.0001; ns, not significant compared with 0 μM BAs LPS+ group. LCA, lithocholic Acid; DCA, deoxycholic acid; HDCA, hyodeoxycholic acid; LPS, Lipopolysaccharide; PMA, Phorbol-12-myristate-13-acetate; TNFA, tumor necrosis factor α; IL-1B, interleukin 1β; NR1H4, nuclear receptor subfamily 1 group H member 4; GPBAR1, G protein-coupled bile acid receptor 1; VDR, vitamin D receptor; NR1H2, nuclear receptor subfamily 1 group H member 2.
