# Supplementary figures and images for: Cholecystectomy-induced secondary bile acids accumulation ameliorates colitis through inhibiting monocyte/macrophage recruitment
Source: Gut Microbes. 2022 Sep 1;14(1):2107387. doi: 10.1080/19490976.2022.2107387 (PMC9450905; doi:10.1080/19490976.2022.2107387)

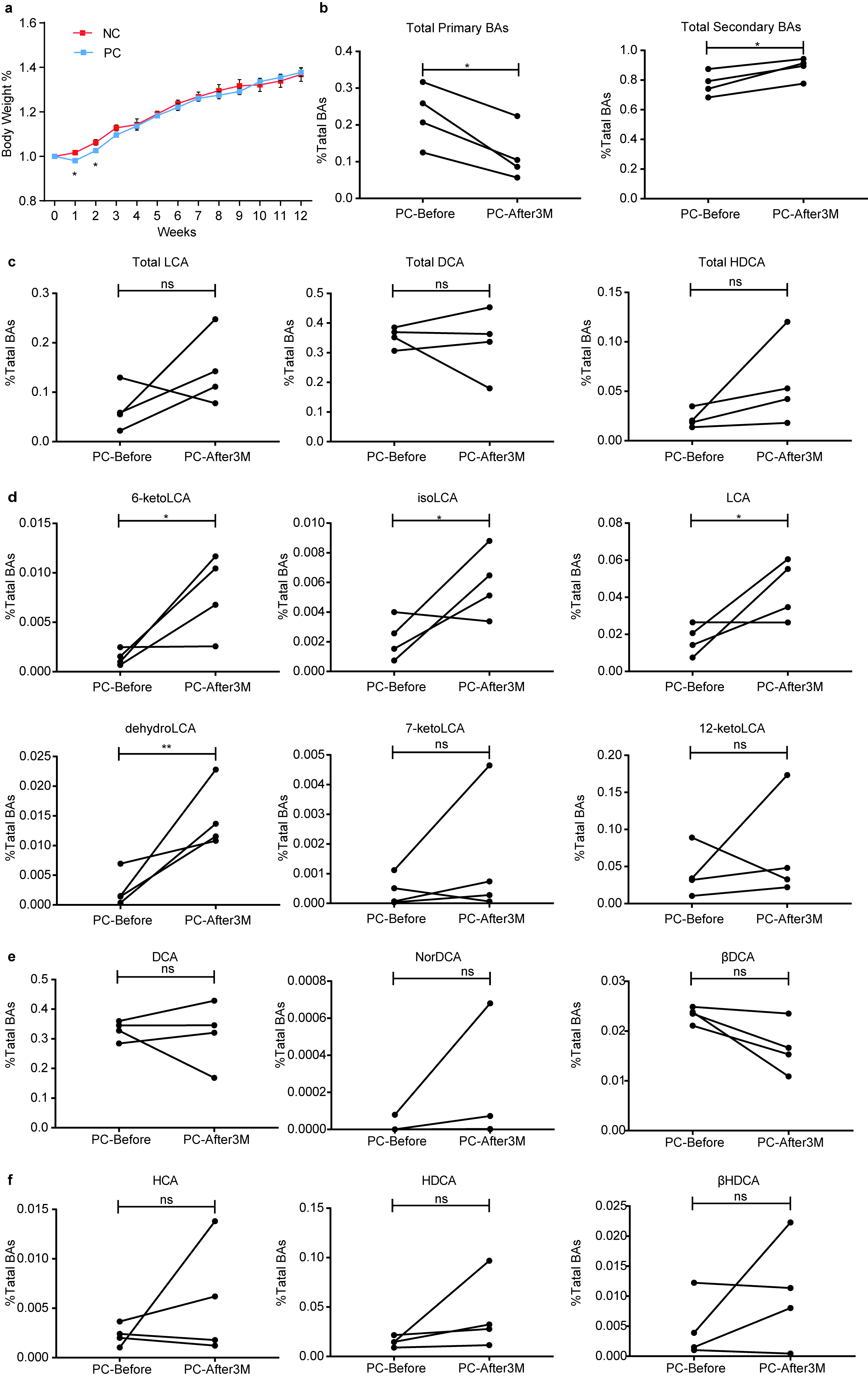

Supplement: Supplemental Material [file KGMI_A_2107387_SM2985.zip › supplementary figure 1 (1).tif]

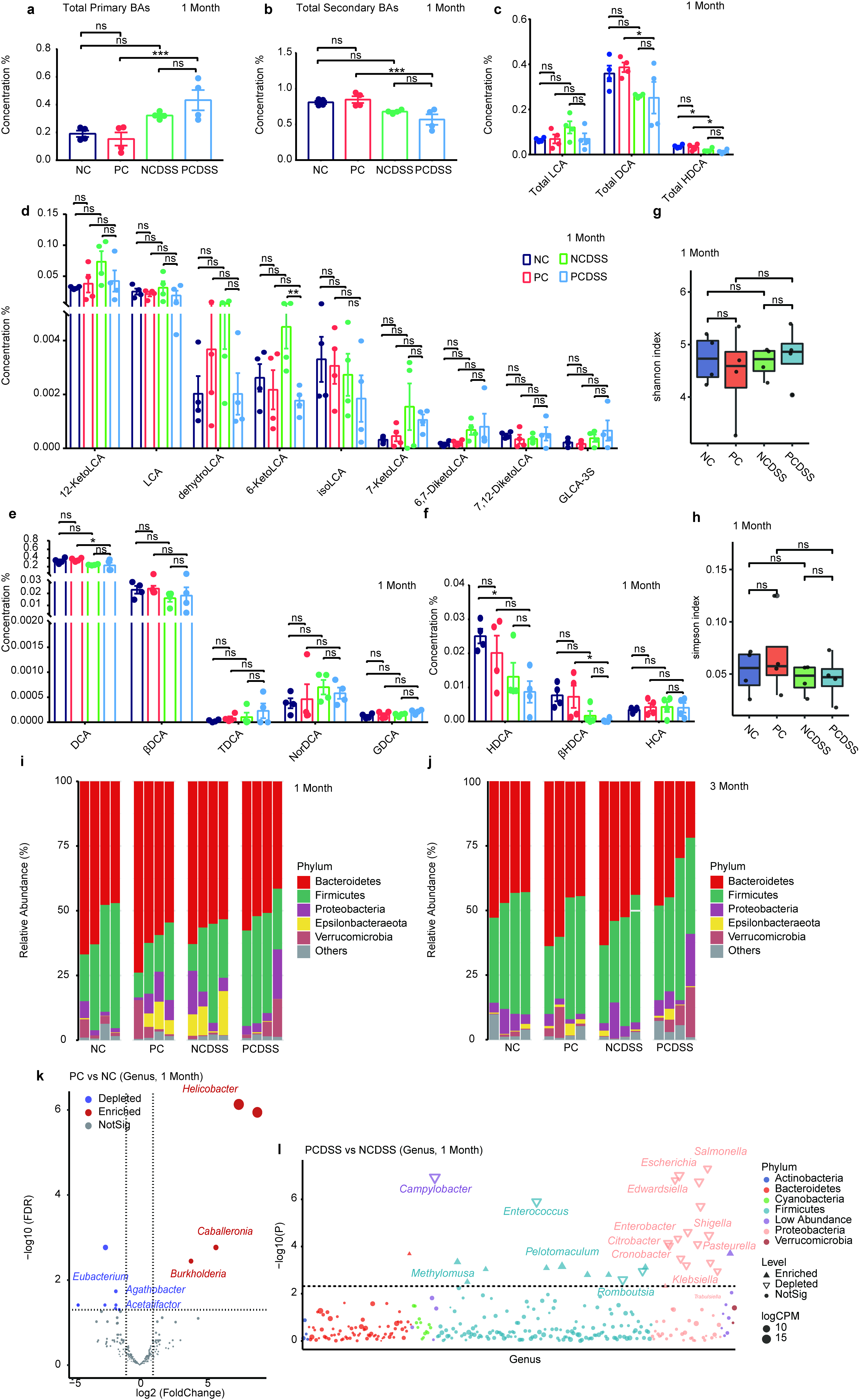

Supplement: Supplemental Material [file KGMI_A_2107387_SM2985.zip › supplementary figure 2 (1).tif]

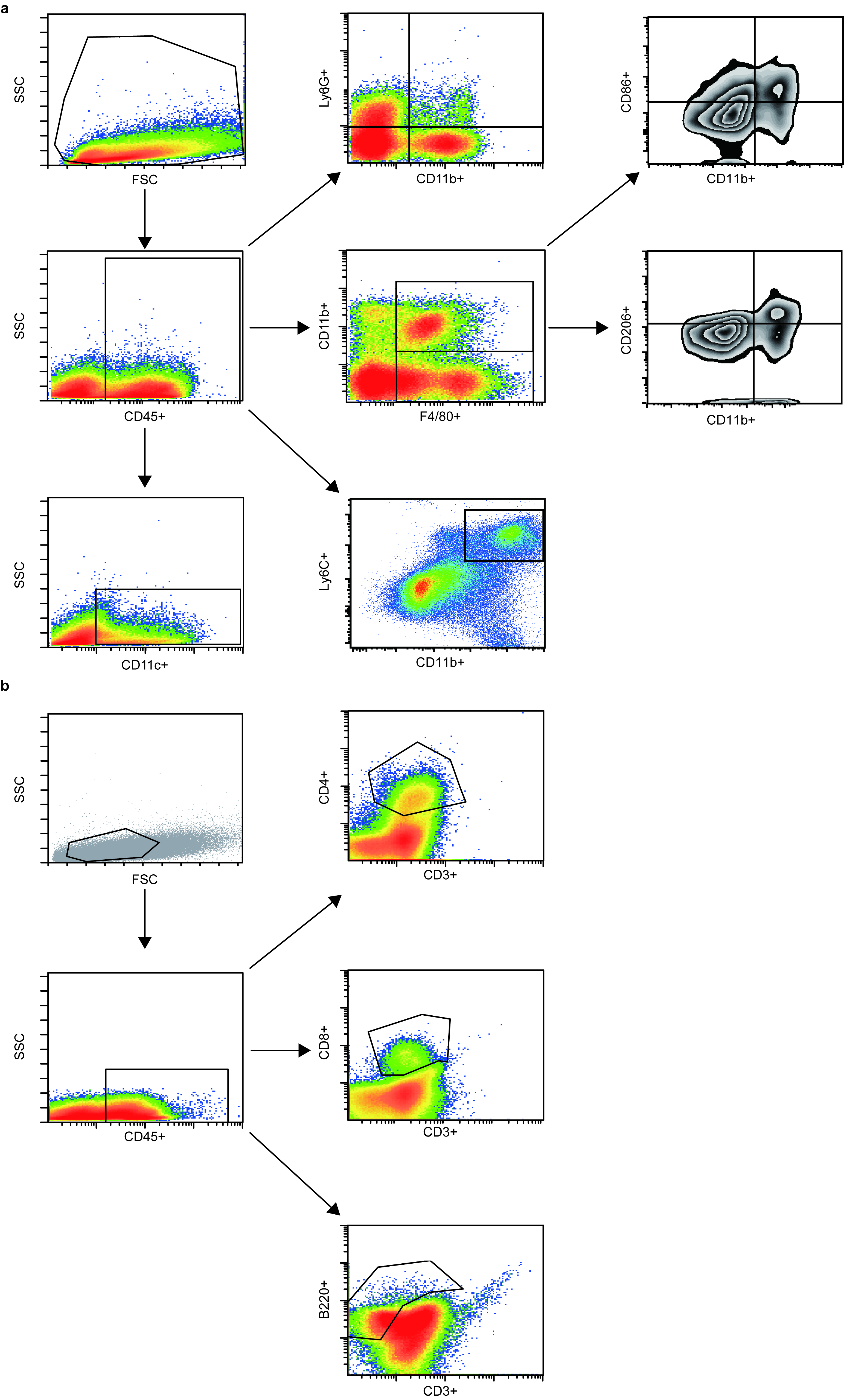

Supplement: Supplemental Material [file KGMI_A_2107387_SM2985.zip › supplementary figure 3 (1).tif]

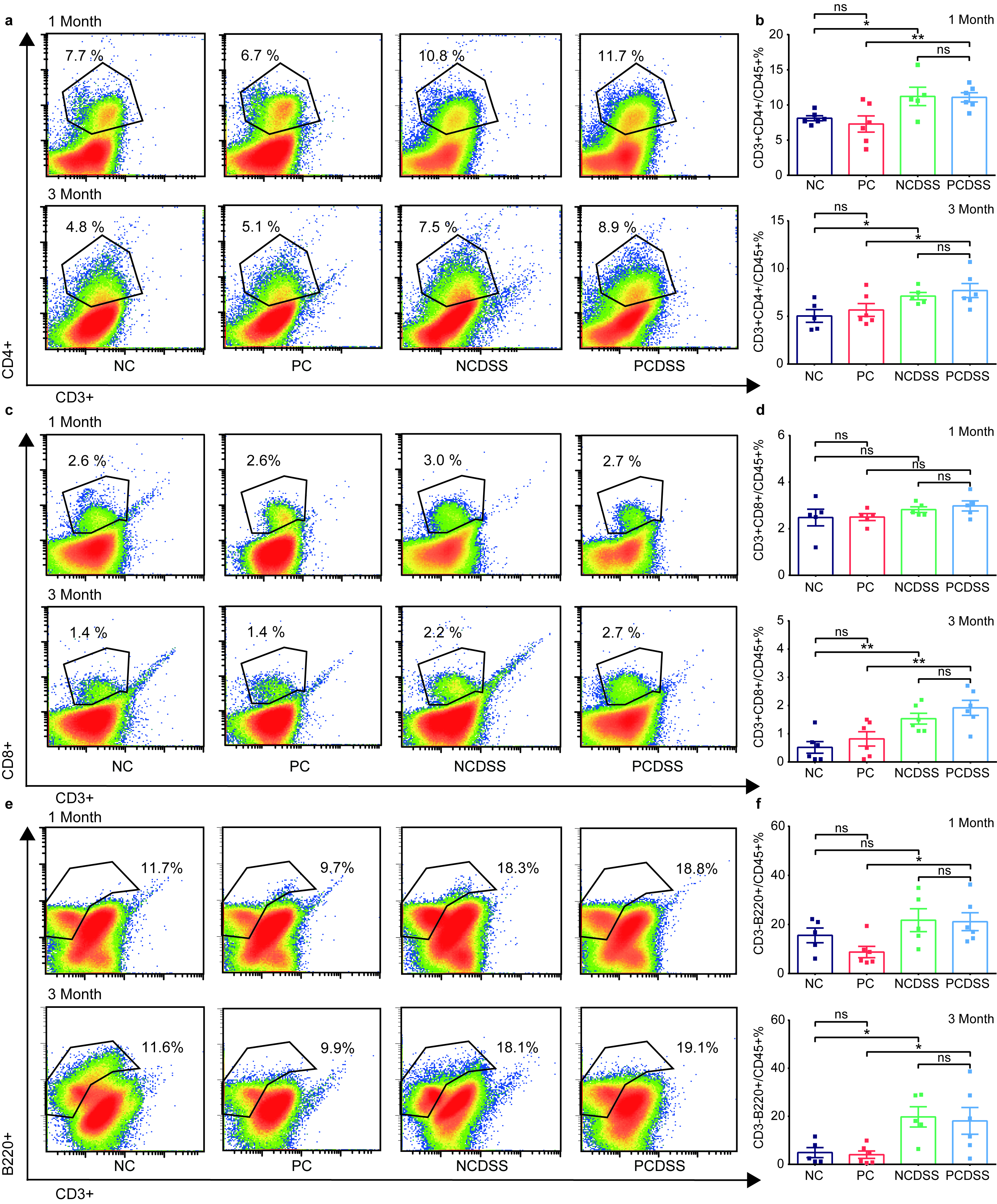

Supplement: Supplemental Material [file KGMI_A_2107387_SM2985.zip › supplementary figure 4 (1).tif]

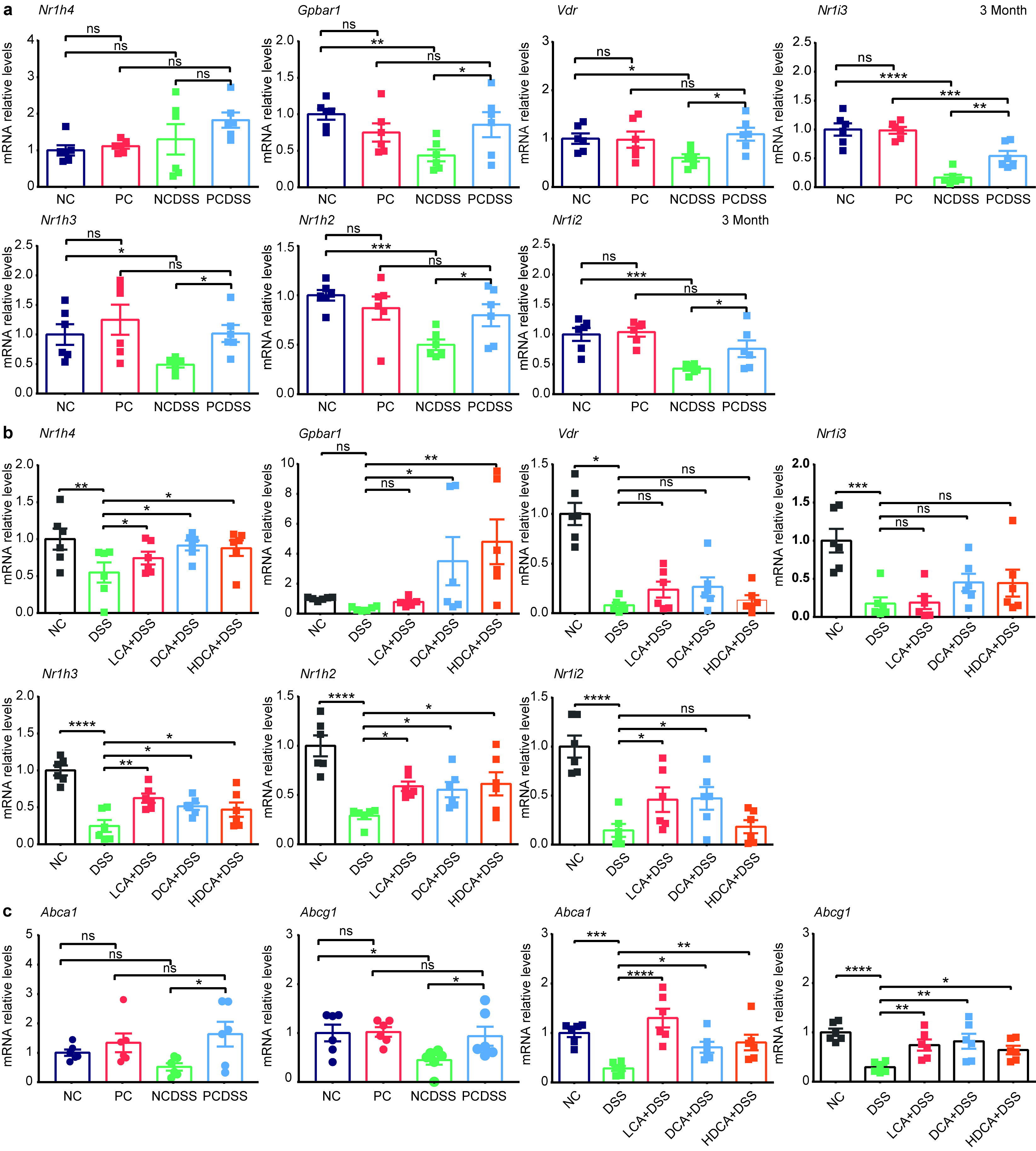

Supplement: Supplemental Material [file KGMI_A_2107387_SM2985.zip › supplementary figure 5 (1).tif]

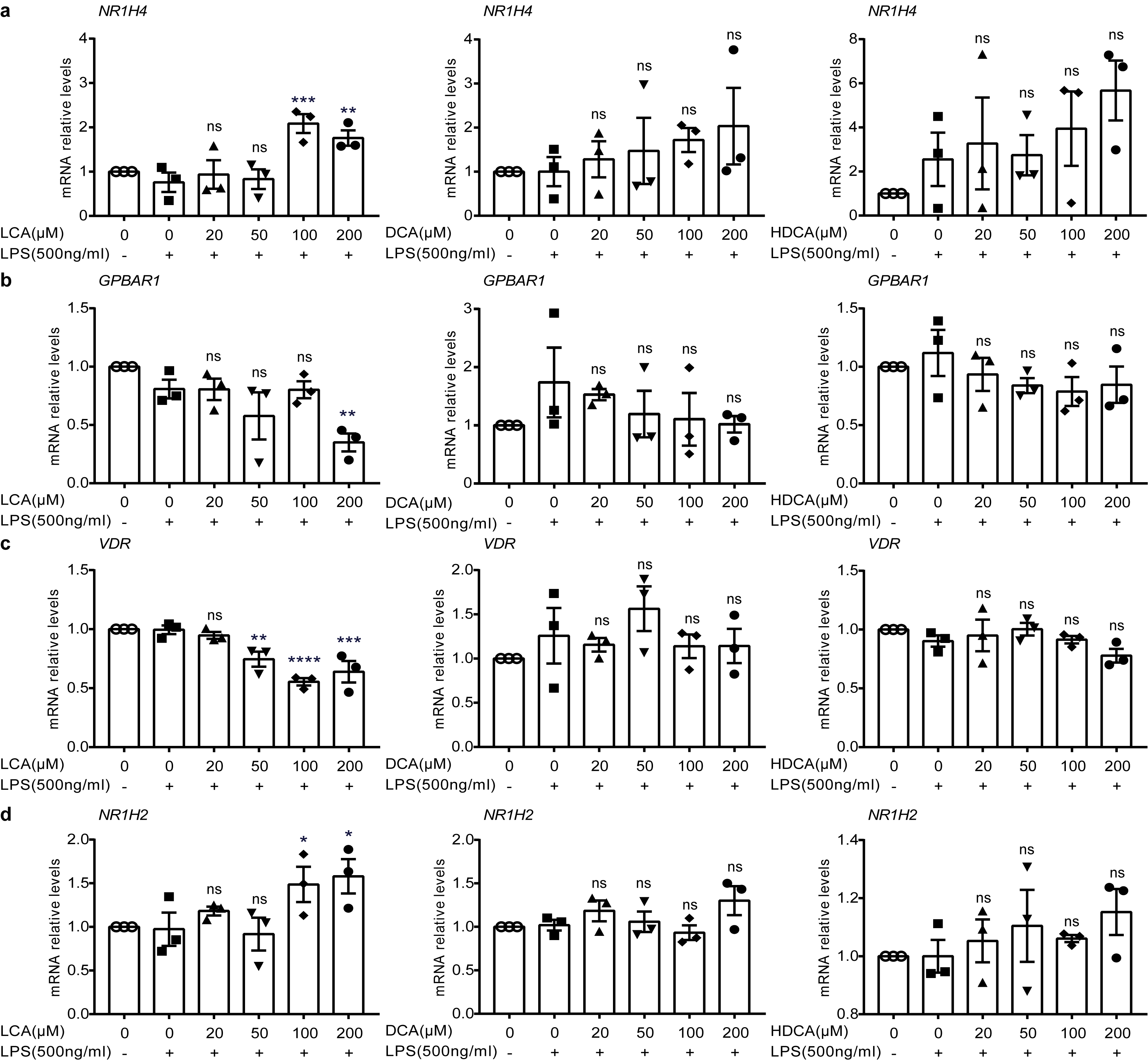

Supplement: Supplemental Material [file KGMI_A_2107387_SM2985.zip › supplementary figure 6 (1).tif]
